# Analysis of Pyroptosis-Related Immune Signatures and Identification of Pyroptosis-Related LncRNA Prognostic Signature in Clear Cell Renal Cell Carcinoma

**DOI:** 10.3389/fgene.2022.905051

**Published:** 2022-06-29

**Authors:** Ming Zhong, Xiaohua Wang, Enyi Zhu, Lian Gong, Lingyan Fei, Liang Zhao, Keping Wu, Chun Tang, Lizhen Zhang, Zhongli Wang, Zhihua Zheng

**Affiliations:** ^1^ Department of Nephrology, Center of Kidney and Urology, The Seventh Affiliated Hospital, Sun Yat-sen University, Shenzhen, China; ^2^ Department of Oncology, Third Xiangya Hospital, Central South University, Changsha, China; ^3^ National Clinical Research Center for Child Health, National Children’s Regional Medical Center, The Children’s Hospital, Zhejiang University School of Medicine, Hangzhou, China; ^4^ Department of Urology, The First Affiliated Hospital of Sun Yat-sen University, Guangzhou, China; ^5^ Department of Internal Medicine and Geriatrics, Zhongnan Hospital, Wuhan University School of Medicine, Wuhan, China

**Keywords:** clear cell renal cell carcinoma, pyroptosis, tumor microenvironment, long noncoding RNA, immune

## Abstract

Clear cell renal cell carcinoma (ccRCC) is a common urinary system malignant tumor with a high incidence and recurrence rate. Pyroptosis is a kind of programmed cell death caused by inflammasomes. More and more evidence had confirmed that pyroptosis plays a very significant part in cancer, and it is controversial whether pyroptosis promotes or inhibits tumors. Consistently, its potential role in ccRCC treatment efficacy and prognosis remains unclear. In this study, we systematically investigated the role of pyroptosis in the ccRCC samples from The Cancer Genome Atlas (TCGA) database. Based on the differentially expressed pyroptosis-related genes (DEPRGs), we identified three pyroptosis subtypes with different clinical outcomes, immune signatures, and responses to immunotherapy. Gene set variation analysis (GSVA), Gene Ontology (GO) analysis, and Kyoto Encyclopedia of Genes and Genomes (KEGG) analysis revealed that pyroptosis activation meant infiltration of more immune cells that is conducive to tumor progression. To further investigate the immunomodulatory effect of pyroptosis in ccRCC, we constructed a pyroptosis-score based on the common differential prognostic genes of the three pyroptosis subtypes. It was found that patients with high pyroptosis-score were in an unfavorable immune environment and the prognosis was worse. Gene set enrichment analysis suggested that immune-related biological processes were activated in the high pyroptosis-score group. Then, the least absolute shrinkage and selection operator (LASSO) Cox regression was implemented for constructing a prognostic model of eight pyroptosis-related long noncoding RNAs (PRlncRNAs) in the TCGA dataset, and the outcomes revealed that, compared with the low-risk group, the model-based high-risk group was intently associated with poor overall survival (OS). We further explored the relationship between high- and low-risk groups with tumor microenvironment (TME), immune infiltration, and drug therapy. Finally, we constructed and confirmed a robust and reliable PRlncRNA pairs prediction model of ccRCC, identified PRlncRNA, and verified it by experiments. Our findings suggested the potential role of pyroptosis in ccRCC, offering new insights into the prognosis of ccRCC and guiding effectual targeted therapy and immunotherapy.

## Introduction

Renal cell carcinoma (RCC) is derived from renal tubular epithelium, accounting for 80%–90% of renal malignant tumors. The incidence of RCC ranks third in urinary system tumors (Sung et al., 2021). Clear cell RCC (ccRCC) is the most common histopathological type of RCC, accounting for about 60%–85% of RCC. Currently, the treatments of ccRCC are multidisciplinary comprehensive treatments including surgery, molecular targeted therapy, immunotherapy, chemotherapy, and radiotherapy. However, ccRCC patients often experience postoperative recurrence, insensitivity to medical treatment, or drug resistance after treatment, leading to poor prognosis, which is related to multiple factors, such as the excessive proliferation of malignant tumor cells and inhibition of cell death. Regulated cell death (RCD) is the defense mechanism against cancer, and it is also a way to drive tumorigenesis, including apoptosis, entosis, necroptosis, pyroptosis, and ferroptosis (Koren and Fuchs, 2021).

Pyroptosis represents an interesting modality of regulated necrosis and is a kind of programmed cell death caused by inflammasomes, which are manifested by the incessant cells swelling until the cells break, leading to the release of cellular contents and intense inflammation. The inflammasome is a key substance in the pyroptosis process. Under the stimulation of pathogens or lipopolysaccharides, it can promote the maturation of the precursor of IL-18 and IL-1β and trigger the pyroptosis process by activating Caspase-1 (Tang et al., 2020; Liu X. et al., 2021b). Current studies have proved that inflammasomes are present in a variety of tumor cells (Dunn et al., 2012). Inflammasome-associated proteins can promote or inhibit the growth of tumor cells in different tumor cells, showing the heterogeneousness of cancer and the complicacy of the immunity microenvironment. Pyroptosis can restrain the proliferation of tumor cells (Nagarajan et al., 2019) and also promote tumor growth by forming an environment suitable for tumor cell growth (Gao et al., 2018). Although there are studies (Jiang et al., 2021; Tang et al., 2021) exploring the potential role of pyroptosis in ccRCC, the data were not well optimized and therefore could not fully explain the effect of pyroptosis on ccRCC. Our study will optimize the data to investigate the influence of pyroptosis on the tumor microenvironment (TME) and drug sensitivity of ccRCC.

Long noncoding RNAs (lncRNAs) are RNA transcripts longer than 200 nucleotides, including natural antisense transcripts, overlapping transcripts, and intronic transcripts. Many studies have shown that lncRNAs have diverse phenotypes and mechanisms by regulating cell proliferation, replication, angiogenesis, cell death, and metastasis (Liu S. J. et al., 2021). LncRNAs have been proved to regulate the pyroptosis of tumor cells. For example, lncRNA-XIST can inhibit pyroptosis to promote non-small cell lung cancer (Liu et al., 2019) and lncRNA ADAMTS9-AS2 restrains gastric cancer and increases the drug sensitivity of cisplatin by promoting pyroptosis (Ren et al., 2020). Pyroptosis-related lncRNA (PRlncRNA) signature has been established as a model to predict the treatment effect and prognoses of various cancers (Hong et al., 2020; Bu et al., 2021; Lv et al., 2021; Ping et al., 2021; Song S. et al., 2021; Tang et al., 2021). However, as a result of the differences in data processing, it is impossible to directly compare the difference in absolute expression levels of lncRNAs among different data sets. Consequently, it is necessary to properly normalize and standardize the expression levels of lncRNAs. Fortunately, the researchers found a way to normalize based on the relative ranking of lncRNAs. For instance, an example of applying these methods was long noncoding RNA pairs (lncRNAPs), which have proved to be reliable (Hong et al., 2020; Song S. et al., 2021).

Given the above, we optimized the data to deeply survey the role of pyroptosis in the TME and targeted therapy effect of ccRCC and integrated a PRlncRNAPs prognostic model that eliminates differences in data processing to forecast the tumor immune infiltration, targeted therapy effect, and prognosis of ccRCC. Finally, we analyzed the lncRNA most related to pyroptosis and verified it by experiments, which provided a promising target for ccRCC.

## Materials and Methods

### Data Sources and Processing

The RNA-seq data (counts value) and the corresponding clinical information of ccRCC from the KIRC project of TCGA_GDC (https://portal.gdc.cancer.gov/), including 539 ccRCCs and 72 normal samples, were downloaded. According to the sample quality annotations provided in The Cancer Genome Atlas (TCGA) database (https://gdc.cancer.gov/about-data/publications/pancanatlas), 100 samples were filtered to exclude patients whose pathological diagnosis was not consistent with ccRCC and who had more than one tumor and underwent radiotherapy and/or chemotherapy (Supplementary Table S1). Finally, we obtained a total of 439 patients for follow-up studies, containing 447 tumor samples and 64 normal samples (some patients had multiple samples; Supplementary Table S2). Then the counts values were converted into TPM values. The gencode.gene.info.v22 file was downloaded from the GDC reference file (https://gdc.cancer.gov/about-data/gdc-data-processing/gdc-reference-files) to do gene annotation and extract lncRNA. Using the “caret” R package, the TCGA cohort was randomly divided into training and validation sets in the ratio 7:3. GSE76207 downloaded from the GEO website (https://www.ncbi.nlm.nih.gov/geo/) was used as the external validation data. Subsequently, 52 pyroptosis-related genes (PRGs) were obtained from MSigDB (REACTOME_PYROPTOSIS) (http://www.broad.mit.edu/gsea/msigdb/) and previously published articles (Supplementary Table S3).

### Consensus Clustering Analysis of DEPRGs

Differential analysis was carried out by the “DESeq2” package (Love et al., 2014), and the counts value downloaded from TCGA was used as the input data (|log2FC| > 1, padj <0.05). The R package “ConsensusClusterPlus” was employed for consensus unsupervised clustering analysis (Wilkerson and Hayes, 2010), dividing patients into different molecular subtypes according to the expression levels of differentially expressed pyroptosis-related genes (DEPRGs). The clustering was based on the following criteria: First, the intragroup correlation was close, while the intergroup correlation was weak. Second, the area under the cumulative distribution function (CDF) curve did not increase significantly. Third, the number of samples in all groups should not be too small. Univariate Cox regression analysis was performed to screen the prognosis-related genes, and the Kaplan–Meier method was used to calculate the overall survival (OS) between different clusters.

### The Differences in Tumor Microenvironment, Immune Infiltration, and Drug Therapy of Pyroptosis Subtypes

The “ESTIMATE” R package was used to evaluate the TME score, such as stromal score, immune score, estimate score, and tumor purity. We evaluated infiltrations of immune cells with “CIBERSORT (Newman et al., 2015),” “XCELL (Aran et al., 2017),” “GSVA (Hänzelmann et al., 2013),” “TIMER (Li et al., 2020),” “QUANTISEQ (Finotello et al., 2019),” “MCPCOUNTER (Becht et al., 2016),” “EPIC(Racle et al., 2017),” and “CIBERSORT-ABS” R packages. Four types of immunophenoscore (IPS), including CTLA4_negative + PD-1_negative, CTLA4_positive + PD-1_negative, CTLA4_negative + PD-1_positive, CTLA4_positive + PD-1_positive, were obtained from the TCIA Database (https://tcia.at/home). The high PD-1_positive IPS showed a well-predicted response to anti-PD-1 treatment. The R package “pRRophetic (Geeleher et al., 2014)" was applied to evaluate the drug sensitivity of first-line targeted therapy for ccRCC, including sunitinib, sorafenib, and axitinib (Numakura et al., 2021). Correlation analysis was performed using the SPEARMAN correlation test and the Wilcoxon signed-rank test.

### Gene Set Enrichment Analysis

To determine whether there were differences in biological processes among different clusters, we downloaded “c2.cp.kegg.v7.4.symbols.gm” and “c5.go.v7.4.symbols.gmt” from MSigDB (http://www.gsea-msigdb.org/gsea/index.jsp). Then, we used “GSVA” package for analysis, and important pathways were shown in the form of heatmap. The “clusterProfiler” package was applied for Gene Ontology (GO) annotation and Kyoto Encyclopedia of Genes and Genomes (KEGG) enrichment pathway analysis of differentially expressed genes (DEGs). An adjusted *p*-value (padj) < 0.05 was considered statistically significant.

### Construction of Prognosis-Related Pyroptosis-Score

We obtained 403 common differential genes through pairwise difference analysis of the three pyroptosis subtypes by the "DESeq2″ R package (|log2FC| > 1, padj <0.01), and then univariate Cox analysis was performed to identify 183 prognosis-related genes. Subsequently, we used the principal component analysis to obtain PC1 and PC2 from feature genes, which were added as the pyroptosis-score of each patient. Then, we compared the survival and clinical characteristics of patients with different pyroptosis-score by Kaplan–Meier analysis. The “estimate,” “CIBERSORT,” and “pRRophetic” packages were utilized to evaluate the immune infiltration and immune microenvironment of patients with different pyroptosis-score, and to preliminarily explore therapeutic drugs. Finally, we used GSEA software for GSEA analysis to analyze the signal pathways that patients with different pyroptosis-score may participate in. The padj <0.05 was defined as statistically significant.

### Construction of a Pyroptosis-Related Long Noncoding RNA Pairs-Based Prognostic Signature

First, we extracted the lncRNAs from the significantly different genes (|log2FC| > 1, padj <0.05) of patients with high or low pyroptosis-score and used co-expression analysis to obtain 76 lncRNAs co-expressed with 19 DEPRGs (|correlation coefficient| > 0.55, *p* < 0.001). These lncRNAs were paired to form PRlncRNAPs, and each PRlncRNAP was scored. Then, we compared the expression levels of these two lncRNAs. If the latter was lower than the former, the score was recorded as 1, otherwise the score was defined as 0. PRlncRNAPs with over 80% or under 20% of score 0 or 1 were excluded from further analysis (Xiong et al., 2020). Subsequently, the PRlncRNAPs were subjected to univariate Cox analysis, and PRlncRNAPs with *p* < 0.001 were used for further study. To address the multicollinearity effect between variables, we used the “glmnet” R package for least absolute shrinkage and selection operator (LASSO) Cox regression (Engebretsen and Bohlin, 2019). Then we constructed a prognostic model by multivariate Cox analysis. Finally, we worked out the riskScore of each sample based on the following formula:

$$\mathrm{riskScore}=\sum_{i=1}^{n}\mathrm{Coe}f_{i}\times X_{i}$$

where *i* means the number of prognostic PRlncRNAPs, Coef is the regression coefficient, and X is the expression value of PRlncRNAPs, respectively. We evaluated the accuracy of riskScore in forecasting the prognoses of patients by the time-dependent receiver operating characteristic (ROC) curve (Heagerty et al., 2000). Then, the median value of riskScore was defined as the cutoff point to divide the patients into prone and low-risk groups in the training or validation group.

### Clinical Value of riskScore

Kaplan–Meier survival analysis was performed to assess the differences in OS of patients with high or low risk. Univariate and multivariate Cox regression analyses were performed on riskScore and clinicopathological features to evaluate whether riskScore was an independent clinical prognostic factor. The hazard ratio (HR) was calculated by “survival” R package. Vesteinn Thomson’s study showed that all TCGA tumors were divided into six immune subtypes, including wound healing, IFN-γ dominant, inflammatory, lymphocyte depleted, immunologically quiet, and TGF-β dominant (Thorsson et al., 2018). Chi-square test was performed to investigate the differences in immune subtypes between high- and low-risk groups.

### Establishment and Validation of a Nomogram Scoring System

The “rms” package was employed for developing a predictive nomogram based on the results of the independent prognosis analysis. In the nomogram scoring system, each variable got a score and the scores of all variables were added to get the total score of each sample. The ability of the model to correctly classify the research event was evaluated by ROC curves and concordance index (C-index). The ROC curves and C-index) were also used to compare the models we built with others (He et al., 2021; Zhang F. et al., 2021; Chen et al., 2022; Sun et al., 2022; Zhang et al., 2022). The differences in the predicted survival events and the virtually observed outcomes were compared by calibration plots of the nomogram. Compared with the ROC curve, decision curve analysis (DCA) considers the clinical utility of a specific model, and we used it to depict the potential clinical effect of the prognostic model (Kerr et al., 2016).

### Clinical Tissue Samples and Cells

The ccRCC tissues and matched adjacent normal tissues were obtained from 32 patients in The First Affiliated Hospital of Sun Yat-sen University (Guangzhou, China). All tissues were immediately frozen in liquid nitrogen and stored at –80°C until RNA was extracted. The samples used in this study were approved by the Ethics Committee of The First Affiliated Hospital of Sun Yat-sen University (Guangzhou, China). The above 32 patients signed the informed consents. HK2, 786-O, 769-P, CAKI-2, and OS-RC-2 in this study were purchased from the Chinese Academy of Science.

### Quantitative Real-Time PCR (qRT-PCR)

Total cellular RNA was extracted using Trizol (Thermofisher Scientific, United States). The RNA was reverse transcribed into cDNA following the steps of the PrimeScrip Reverse Transcription Kit (Takara, Dalian, China). The PCR reaction system was configured and analyzed according to the instructions of SYBR Green Pro Taq HS premix (Accurate Biology, Changsha, China). The PCR primer sequences were: AC002331.1 forward: 5′-TGC​TGC​CAA​AGT​AGG​AGG​ATT​C-3′, reverse: 5′-GAA​GGA​AGT​GCT​CCA​CAC​AGT​C-3'; GAPDH forward: 5′-GTC​TCC​TCT​GAC​TTC​AAC​AGC​G-3′, reverse: 5′-ACC​ACC​CTG​TTG​CTG​TAG​CCA​A-3'.

### Statistical Analyses

The unpaired *t*-test and Wilcoxon rank-sum test were used to evaluate the difference between normally distributed and non-normally distributed data, respectively. OS curves were obtained with Kaplan–Meier analysis and differences between groups were calculated with log-rank test. R software (version 4.0.3) and Adobe Illustrator (version 25.0) were employed for statistical analysis and drawing. *p*-value <0.05 indicates statistical significance.

## Results

### Data Processing

This study was conducted according to the flow chart (Supplementary Figure S1). In order to make our research more trustworthy, TCGA data were randomly divided into a training set (*n* = 308) and a validation set (*n* = 131) in the ratio 7:3. There was no difference in various clinicopathological parameters between the two datasets, with *p*-value > 0.05 (Table 1).

**TABLE 1:** Clinicopathological features of 439 ccRCC patients.

Type	Details	Total	Training	Validation	*p* value
Age	≤65	293 (66.74%)	201 (65.26%)	92 (70.23%)	0.3679
	>65	146 (33.26%)	107 (34.74%)	39 (29.77%)	—
Gender	Female	159 (36.22%)	111 (36.04%)	48 (36.64%)	0.9907
	Male	280 (63.78%)	197 (63.96%)	83 (63.36%)	—
Fustat	Alive	300 (68.34%)	211 (68.51%)	89 (67.94%)	0.9961
	Dead	139 (31.66%)	97 (31.49%)	42 (32.06%)	—
Grade	G1	13 (2.96%)	9 (2.92%)	4 (3.05%)	0.4889
	G2	180 (41%)	133 (43.18%)	47 (35.88%)	—
	G3	176 (40.09%)	117 (37.99%)	59 (45.04%)	—
	G4	63 (14.35%)	43 (13.96%)	20 (15.27%)	—
	Unknown	7 (1.59%)	6 (1.95%)	1 (0.76%)	—
Stage	Stage I	208 (47.38%)	145 (47.08%)	63 (48.09%)	0.4305
	Stage II	46 (10.48%)	37 (12.01%)	9 (6.87%)	—
	Stage III	109 (24.83%)	75 (24.35%)	34 (25.95%)	—
	Stage IV	73 (16.63%)	49 (15.91%)	24 (18.32%)	—
	Unknown	3 (0.68%)	2 (0.65%)	1 (0.76%)	—
T	T1	214 (48.75%)	150 (48.7%)	64 (48.85%)	0.1673
	T2	56 (12.76%)	46 (14.94%)	10 (7.63%)	—
	T3	160 (36.45%)	106 (34.42%)	54 (41.22%)	—
	T4	9 (2.05%)	6 (1.95%)	3 (2.29%)	—
M	M0	339 (77.22%)	242 (78.57%)	97 (74.05%)	0.6895
	M1	69 (15.72%)	47 (15.26%)	22 (16.79%)	—
	Unknown	31 (7.06%)	19 (6.17%)	12 (9.16%)	—
N	N0	196 (44.65%)	138 (44.81%)	58 (44.27%)	0.7167
	N1	13 (2.96%)	8 (2.6%)	5 (3.82%)	—
	Unknown	230 (52.39%)	162 (52.6%)	68 (51.91%)	—

### Identification of Pyroptosis Subtypes in ccRCC

**FIGURE 1:**
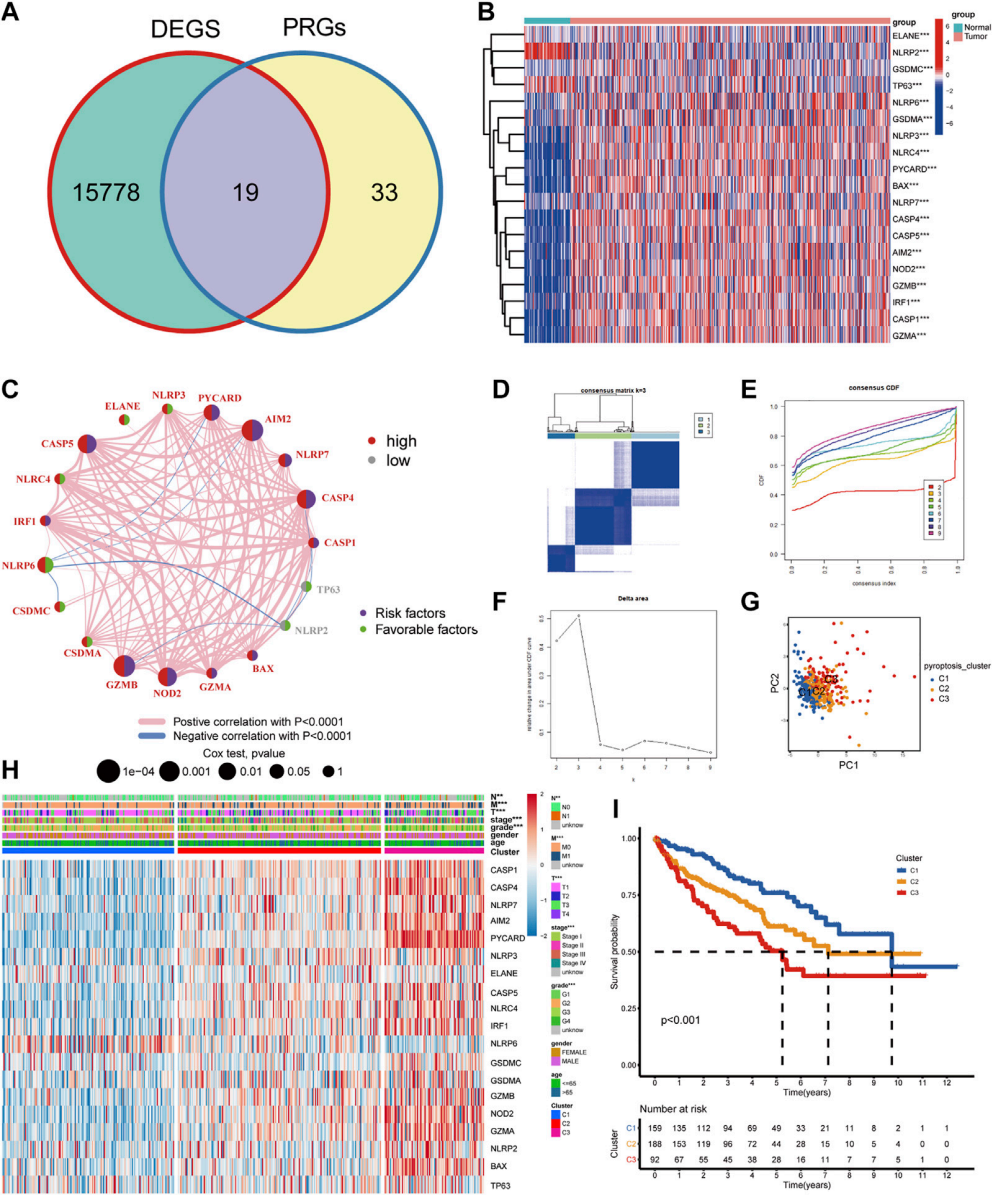
The landscape of DEPRGs in ccRCC and identification of pyroptosis subtypes. **(A)** The Venn diagram showed overlapping genes. **(B)** A heatmap was used to show the differential expression of 19 DEPRGs expressions in ccRCC and noncancerous tissues. **(C)** Interaction between DEPRGs in ccRCC. The line connecting DEPRGs represented their interaction, and the strength of the correlation between DEPRGs was indicated by the thickness of the line. Pink and blue, respectively, represented positive and negative correlations. The color of the left half circle represented the gene expression in ccRCC, with high expression in red and low in gray. The color of the right half circle represented the influence of genes on prognosis. Purple was the risk factor of prognosis, green was the favorable factor of prognosis, and the size of the circle represented the *p*-value. **(D)** Consensus matrix when k was 3. **(E)** Consensus CDF when k was between 2 and 9. **(F)** Delta area showed the relative change of the area under the CDF curve comparing k and k−1 and it met our screening criteria when k = 3. **(G)** PCA showed marked differences in the transcriptome among the three pyroptosis subtypes. **(H)** The clinicopathological characteristics of the three subtypes and different expression levels of DEPRGs. **(I)** Survival curves of patients with three pyroptosis subtypes (***p < 0.001, **p < 0.01, *p < 0.05).

First, we obtained the DEGs by investigating the difference between the ccRCC group and the normal group using the “DESeq2” package. Then, 19 DEPRGs were acquired after the intersection with PRGs (Figure 1A), including 17 upregulated and two downregulated DEPRGs (Figure 1B). Univariate Cox regression was performed to reveal the prognosis of 19 DEPRGs in patients with ccRCC (Supplementary Table S4). *p* < 0.05 was utilized as the screening threshold to screen 14 genes related to prognosis. Then, we divided patients into high and low groups based on the optimal cutoff value of the above 14 genes, and the survival analysis curves of 14 genes were obtained (Supplementary Figure S2). The pyroptosis network showed the interaction and prognostic value of the 19 DEPRGs (Figure 1C). Then, univariate Cox analysis was performed to identify seven prognosis-related DEPRGs. The “ConsensusClusterPlus” package was applied to cluster the TCGA-KIRC cohort into different groups through the consistent expression of the seven DEPRGs. When the consensus matrix k value was 3, the crossover among ccRCC samples was the least, which met our screening criteria (Figure 1D–F). PCA demonstrated that the three subtypes were distributed in different clusters (Figure 1G). There were significant differences in clinicopathological parameters such as grade, stage, T, M, and N of the three pyroptosis subtypes of patients (Figure 1H). Kaplan–Meier survival analysis suggested that patients with different pyroptosis subtypes had significant differences in OS (*p* < 0.001), among which patients with C1 subtype had the best OS and C3 subtype was the worst (Figure 1I).

### Immune Landscape and Drug Response of Pyroptosis Subtypes

**FIGURE 2:**
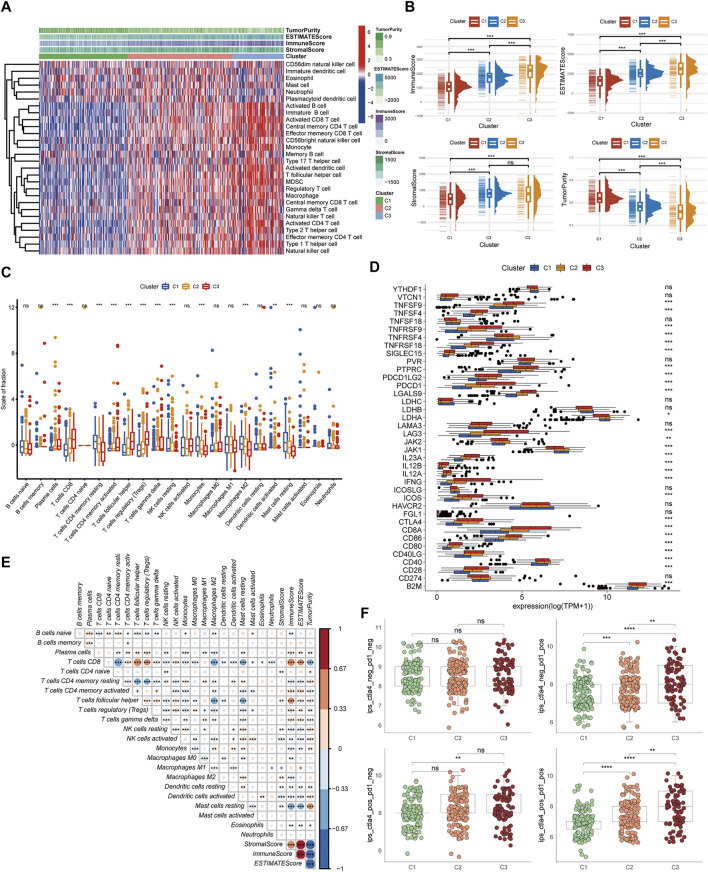
Immune landscape and drug response of pyroptosis subtypes. **(A)** The heatmap of immune cell infiltration annotations and immune microenvironment scores in pyroptosis subtypes. **(B)** The levels of estimate score, immune score, stromal score, and tumor purity in pyroptosis subtypes. **(C)** The relative abundance of 22 immune cell types in pyroptosis subtypes. **(D)** The expression levels of common immune checkpoints among pyroptosis subtypes. **(E)** The heatmap of the interaction between immune cells. **(F)** IPS comparison among the three pyroptosis subtypes of the patients with ccRCC in the CTLA4 negative/positive or PD-1 negative/positive groups. CTLA4_positive or PD-1_positive, respectively, stood for anti-CTLA4 or anti-PD-1 therapy (***p < 0.001, **p < 0.01, *p < 0.05).

**FIGURE 3:**
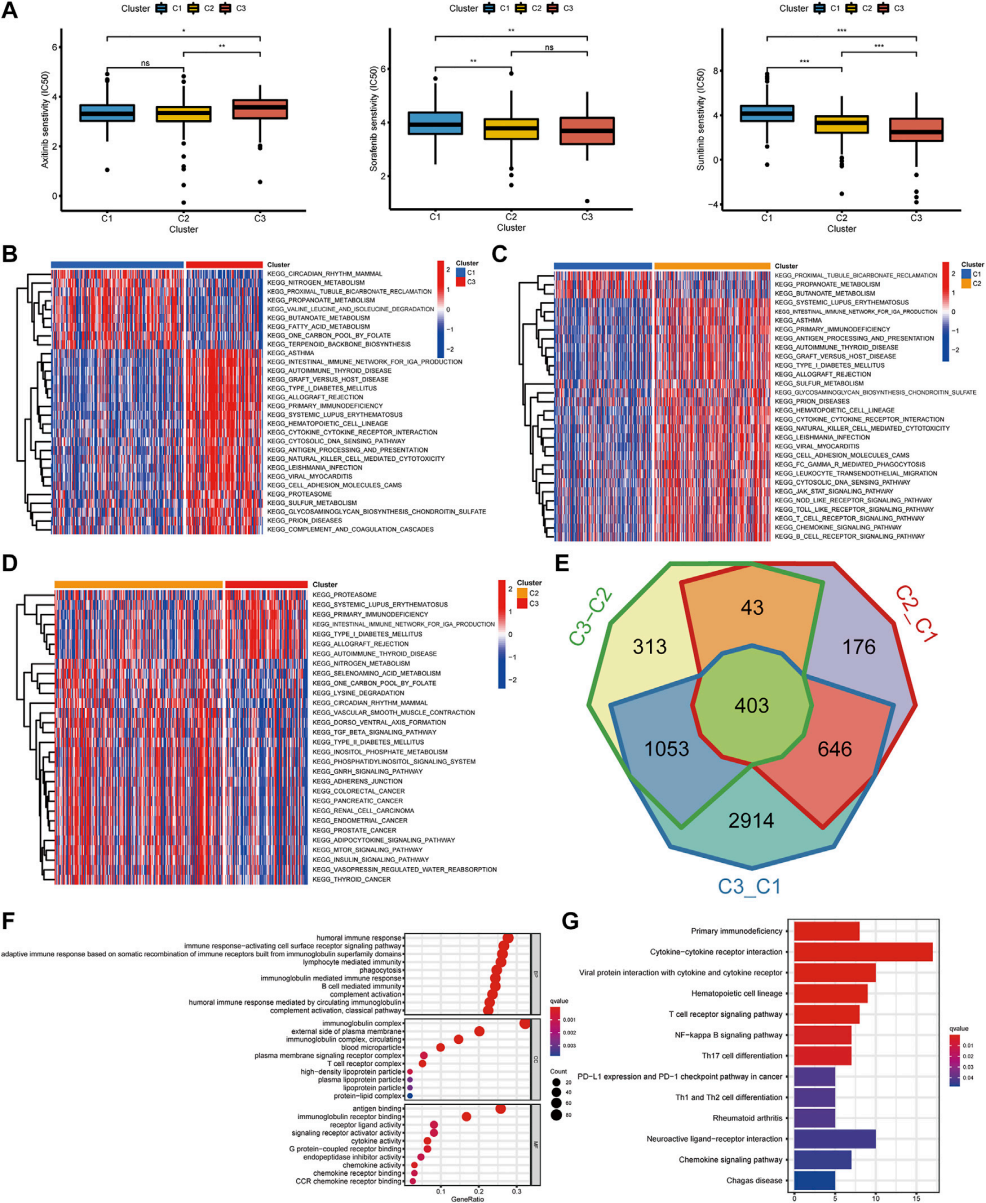
The interaction and correlation among the pyroptosis subtypes. **(A)** The different IC50 of targeted drugs in the three pyroptosis subtypes. **(B–D)** GSVA enrichment analysis presents the activation states of biological pathways in the three pyroptosis subtypes. The heatmaps were utilized to visualize the biological processes. Red and blue, respectively, represented the activated and inhibited pathways. **(E)** A Venn diagram revealed the common differential genes of the three pyroptosis subtypes. **(F,G)** GO and KEGG analysis of DEGs in the three pyroptosis subtypes (***p < 0.001, **p < 0.01, *p < 0.05).

To further investigate the relationship between pyroptosis subtypes and immune cells, we quantified the enrichment scores of single-sample gene set enrichment analysis (ssGSEA) for different immune cell subgroups. The results indicated that compared with subtype C1, activated dendritic cell, MDSC, macrophage, activated CD4 T cell, T follicular helper cell, activated CD8 T cell, and natural killer T cell infiltrated more in C2 and C3 (Figure 2A). Then “ESTIMATE” package was utilized to evaluate the TME scores of the three subtypes, containing stromal score, immune score, estimate score, and tumor purity. Compared with C1, C2 and C3 subtypes showed that the tumor purity was lower and immune cells and stromal cells were higher. Estimate score suggested that the relative content of stromal cells and immune cells in C3 subtype was the highest (Figure 2B). CIBERSORT was employed for analyzing the relative abundance of 22 immune cells in each tumor sample, and the results suggested that there were more T cells regulatory (Tregs), T follicular helper cells, and CD8 T cells in C3 subtype (Figure 2C). Currently, immune checkpoint inhibitors are the first-line therapeutic drugs for advanced ccRCC. We had screened several targeted biomarkers that were essential for immunotherapy and further clarified whether the pyroptosis subtypes were related to them. We discovered that CTLA4, PDCD1, and PDCD1LG2 had the highest expression in the C3 subtype (Figure 2D). Survival analysis revealed that when FGL1, LAG3, TNFRSF18, and IL-23A were highly expressed, the prognosis of patients was worse, while JAK1, JAK2, and LDHA were on the contrary (Supplementary Figure S3), which may provide a new target for immunotherapy of ccRCC. The heatmap exhibiting the interaction of immune cells was plotted, which displayed that the immune score was highly negatively correlated with different types of macrophages but positively correlated with different types of T cells (Figure 2E). We performed immunophenogram analysis for analyzing the relationship between IPS and pyroptosis subtypes (Figure 2F). The outcomes revealed that in CTLA4_negative + PD-1_positive type and CTLA4_positive + PD-1_positive type, the IPS of C3 subtype was the highest. These results suggested that C3 subtype patients were more sensitive to anti-PD-1 therapy or a combination of anti-PD-1 and anti-CTLA4 therapies. The targeted therapies, including axitinib, sorafenib, and sunitinib, were the first-line therapeutic drugs for ccRCC. We explored the effect of pyroptosis subtypes on the sensitivity of these drugs in ccRCC (Figure 3A). Interestingly, we realized that the IC50 of axitinib was higher in C3 subtype, while those of sorafenib and sunitinib were lower. Based on the above analysis, we found that the state of pyroptosis may significantly inhibit or enhance the expression of specific immune cell types and then potentially affect the reaction to immunotherapy and targeted therapies.

### Interaction and Correlation Between Pyroptosis Subtypes

The gene set variation analysis (GSVA) enrichment analysis was used for investigating the potential biological processes among the three pyroptosis subtypes (Figure 3B–D). It was found that metabolism-related pathways were significantly enriched in C1 subtypes, such as KEGG_FATTY_ACID_METABOLISM, while C2 and C3 subtypes presented enrichment pathways related to complete immune activation, including KEGG_ANTIGEN_PROCESSING_AND_PRESENTATION, KEGG_PRIMARY_IMMUNODEFICIENCY, KEGG_CYTOKINE_CYTOKINE_RECEPTOR_INTERACTION, KEGG_ANTIGEN_PROCESSING_AND_PRESENTATION, KEGG_T_CELL_RECEPTOR_SIGNALING_PATHWAY, and KEGG_TOLL_LIKE_RECEPTOR_SIGNALING_PATHWAY. To further clarify the biological differences between the three pyroptosis subtypes, 403 common difference genes were obtained by pairwise difference analysis between pyroptosis subtypes (Figure 3E), and genes function enrichment analysis was carried out (Figure 3F). It was found that the common difference genes were significantly enriched in immune-related biological processes. KEGG enrichment pathway analysis further confirmed the activation of immune-related pathways, including cytokine–cytokine receptor interaction, T-cell receptor signaling pathway, and chemokine signaling pathway (Figure 3G). It suggested that pyroptosis played a vital role in the immune regulation of TME.

### Construction of Pyroptosis-Score and the Relationship With TME and Drug Response

**FIGURE 4:**
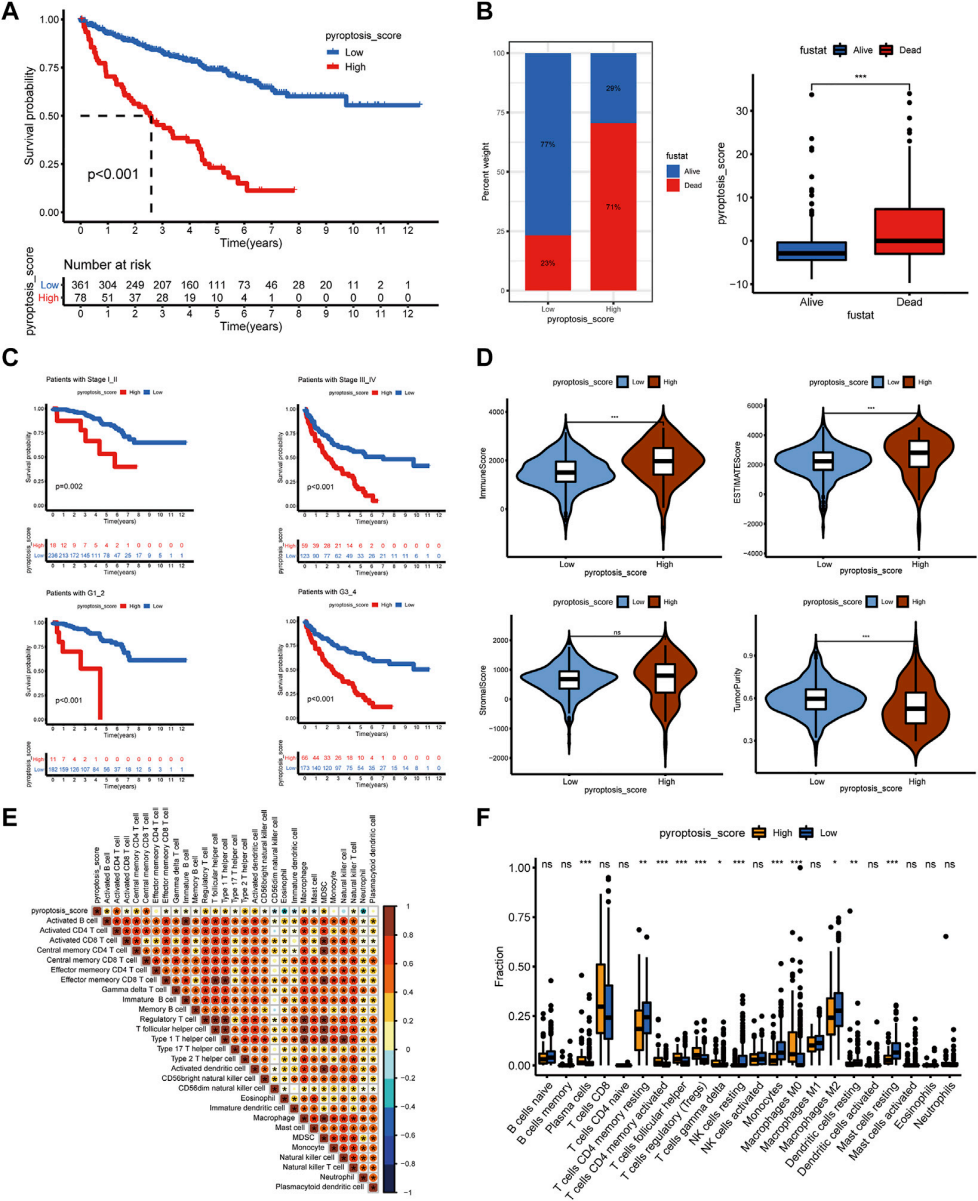
The clinical characteristics and immune landscape of the pyroptosis-score groups. **(A)** Survival curves of patients with high and low pyroptosis-scores. **(B)** The ratio of survival status in patients with high and low pyroptosis-scores and the difference in pyroptosis-score level of different survival status. **(C)** The survival curves of patients with high and low pyroptosis-scores at different stages and grades. **(D)** The different levels of estimate score, immune score, stromal score, and tumor purity in high and low pyroptosis-score groups. **(E)** Correlation analysis between pyroptosis-score and immune cells. **(F)** Differences in relative abundance of 22 immune cell types in patients with high and low pyroptosis-scores (***p < 0.001, **p < 0.01, *p < 0.05).

**FIGURE 5:**
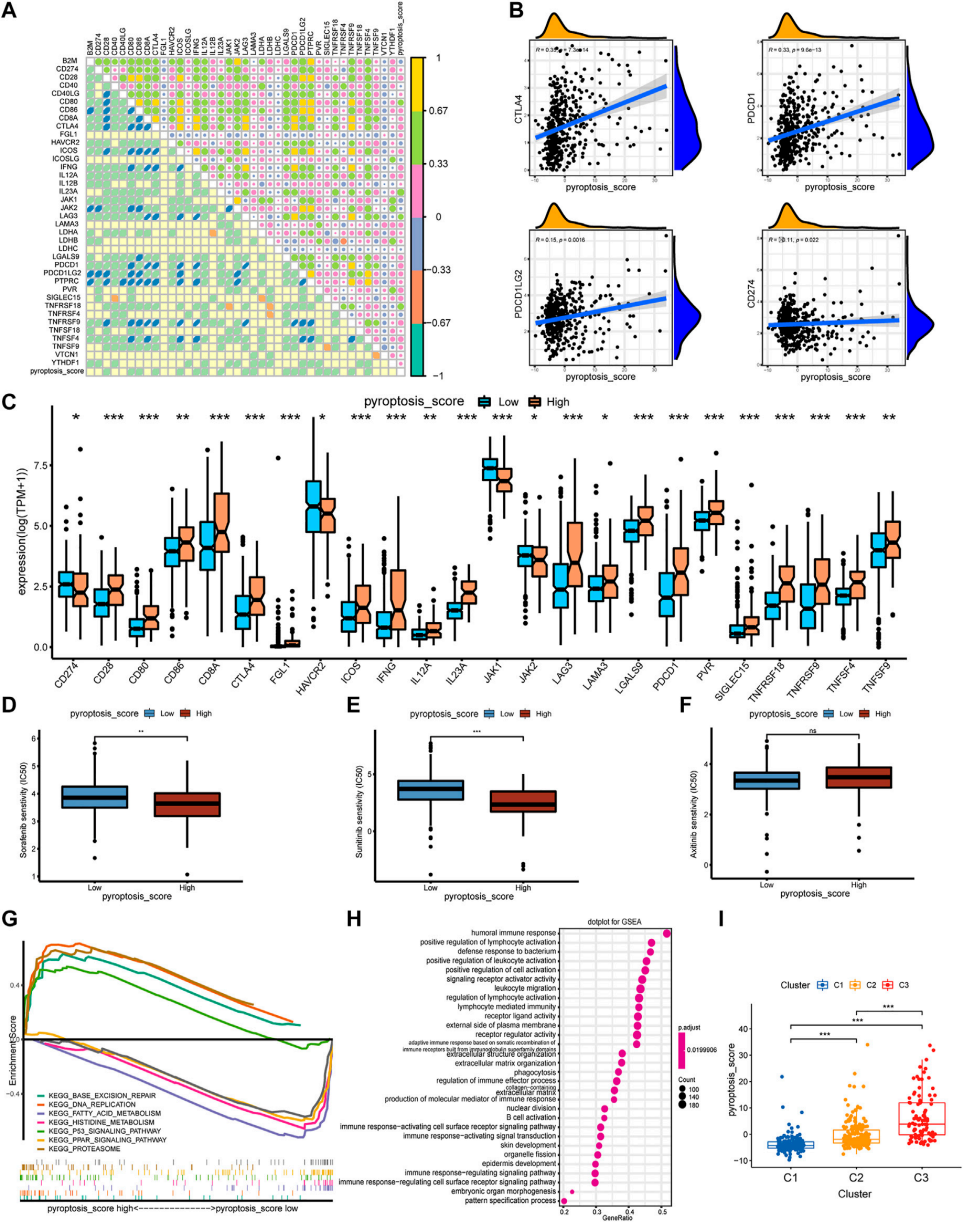
The correlation of pyroptosis-score with immune checkpoints and drug response, and the biological process of grouping. **(A)** Spearman correlation coefficient diagram of pyroptosis-score and common immune checkpoints. **(B)** Spearman correlation scatter plot between pyroptosis-score and CTLA4, PDCD1, PDCD1LG2, and CD274. **(C)** The different expression levels of common immune checkpoints between the high and low pyroptosis-score groups. **(D–F)** The different IC50 of the targeted drugs among high and low pyroptosis-score. **(G,H)** GSEA enrichment analysis of high and low pyroptosis-score groups, including KEGG pathways **(G)**, and GO annotation **(H)** (***p < 0.001, **p < 0.01, *p < 0.05). **(I)** The pyroptosis-score of the three pyroptosis subtypes.

To further understand the immune regulation of pyroptosis on ccRCC, we made an effort to analyze the common differential genes of the three pyroptosis subtypes and established a pyroptosis-score based on the subtype-related DEGs for each patient. After univariate Cox regression analysis, 183 prognosis-related genes were obtained from 403 common DEGs of pyroptosis subtypes, and then PCA was performed. We add the scores of PC1 and PC2 to get the pyroptosis-score of each sample. After that, the optimal threshold was obtained by the R package “survminer” to divide the patients into high and low pyroptosis-score groups. Relative to the low pyroptosis-score patients, there were a shorter OS (Figure 4A) and more deaths (Figure 4B) in the high group. Kaplan–Meier analysis was applied to investigate the relationship between pyroptosis-score and the prognosis in different clinical groups. The results indicated that regardless of the patient’s stage and grade, the prognosis of patients in the high pyroptosis-score group was worse (Figure 4C). Relative to low pyroptosis-score, patients in the high group had lower tumor purity and higher immune cells and stromal cells. The estimate score also revealed that the relative content of stromal cells and immune cells in the high pyroptosis-score group was higher (Figure 4D). Correlation analysis indicated that pyroptosis-score was positively correlated with activated CD4 T cell, activated CD8 T cell, T follicular helper cell, type 2 T-helper cell, activated dendritic cell, and MDSC (Figure 4E). Compared with the low pyroptosis-score group, the patients in the high group infiltrated more memory-activated CD4 T cell, T follicular helper cells, Tregs, and macrophage M0 cells (Figure 4F). Correlation analysis between scores and immune checkpoints presented that pyroptosis-score was positively correlated with CTLA4, PDCD1, and PDCD1LG2 (Figure 5A,B). The high pyroptosis-score group expressed higher CTLA4 and PDCD1 and lower CD274 (Figure 5C). Then, we performed immunophenogram analysis for analyzing the relationship between IPS and pyroptosis-score (Supplementary Figure S4). The outcomes revealed that in CTLA4_negative + PD-1_positive type, CTLA4_positive + PD-1_negative type, and CTLA4_positive + PD-1_positive type, the IPS of high pyroptosis-score group was higher. These results suggested that patients in high pyroptosis-score groups were more sensitive to immunity therapies. In terms of targeted drug treatment sensitivity, the high pyroptosis-score group was more sensitive to sorafenib and sunitinib treatment (Figure 5D,E), but there was no significant difference in axitinib (Figure 5F). GSEA enrichment analysis indicated that the pathways of the high pyroptosis-score group were mainly related to DNA damage repairs, such as KEGG_BASE_EXCISION_REPAIR, KEGG_DNA_REPLICATION, and KEGG_P53_SIGNALING_PATHWAY. The pathways of the low pyroptosis-score group were enriched in the activation of metabolism pathways such as KEGG_FATTY_ACID_METABOLISM, KEGG_HISTIDINE_METABOLISM, and KEGG_TRYPTOPHAN_METABOLISM (Figure 5G). We also found that immune-related biological processes were activated in the high pyroptosis-score group (Figure 5H). There were significant differences in the pyroptosis-score among the pyroptosis subtypes. The outcomes revealed that the pyroptosis-score was the lowest in C1 and the highest in C3 (Figure 5I). The characteristics of patients with C3 subtype and high pyroptosis-score group were consistent. Both have immune activation characteristics, and the prognosis was worse than that in other groups.

### Identification of OS-Related PRlncRNAPs and Establishment of the Prognostic Model

**FIGURE 6:**
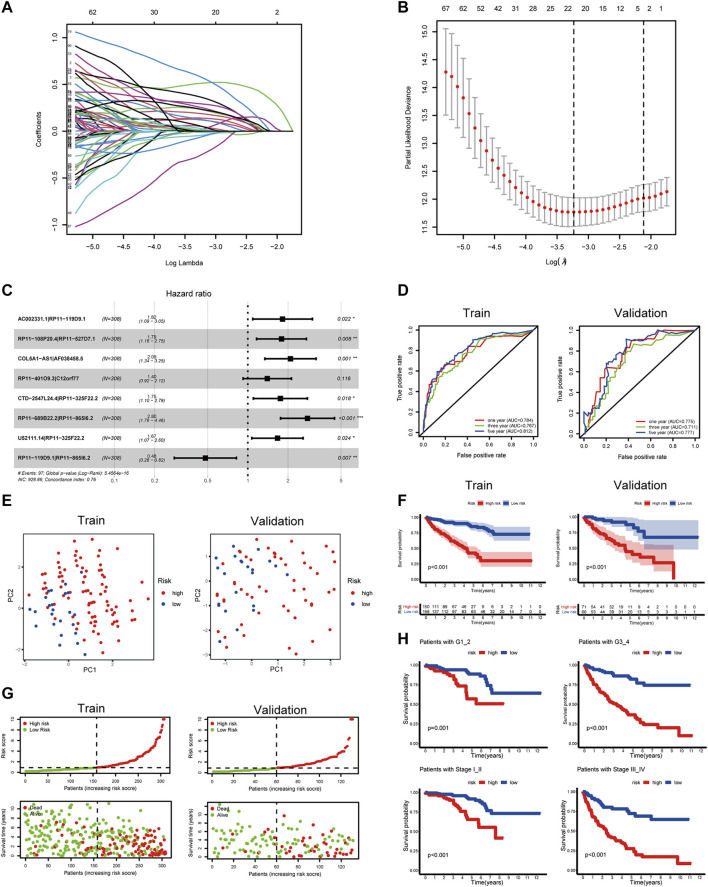
Construction of a prognostic signature for ccRCC. **(A,B)** LASSO coefficient plot of PRlncRNAPs. **(C)** Forest plots of the eight OS-related PRlncRNAPs. **(D)** The ROC curve showed the diagnostic value of riskScore for the ccRCC prognosis. **(E)** PCA on the basis of prognostic characteristics. The red and blue dots, respectively, represented patients with high and low risk. **(F)** OS analysis of patients with high and low riskScore. **(G)** Distribution of riskScore and survival status of patients. **(H)** Survival curves of patients with the high- and low-risk groups in different grades and stages (***p < 0.001, **p < 0.01, *p < 0.05).

There were 76 lncRNAs co-expressed with 19 DEPRGs in the differential genes of the high and low pyroptosis-score groups. By recombining the 76 lncRNAs, we obtained 1,425 PRlncRNAPs and the corresponding relative expression levels; then 90 PRlncRNAPs related to prognosis were recognized through the univariate Cox regression analysis (*p* < 0.001). Subsequently, we extracted 22 OS-related PRlncRNAPs after 1,000 iterations by LASSO Cox regression analysis (Figure 6A,B). Ultimately, we obtained eight PRlncRNAPs to construct the risk model by multivariate regression analysis (Figure 6C), and the coefficient of PRlncRNAPs was employed for calculating the riskScore (Supplementary Table S5). The above method was carried out in the randomly selected training set, and the same coefficient was applied to the validation set. The ROC curves showed that the riskScore exhibited excellent prediction ability. In the training set, the 1-, 3-, and 5-year areas under the curve (AUC) were 0.784, 0.767, and 0.812, respectively. The AUCs in the validation set were, respectively, 0.775, 0.711, and 0.777 (Figure 6D). Based on the median value of riskScore in the training set, the patients with ccRCC were divided into high- and low-risk groups. PCA displayed distinguishable dimensions between the high- and low-risk groups in the training set while it did not seem to be so perfect in the validation set (Figure 6E).

### Clinical Value of Risk Groups

**FIGURE 7:**
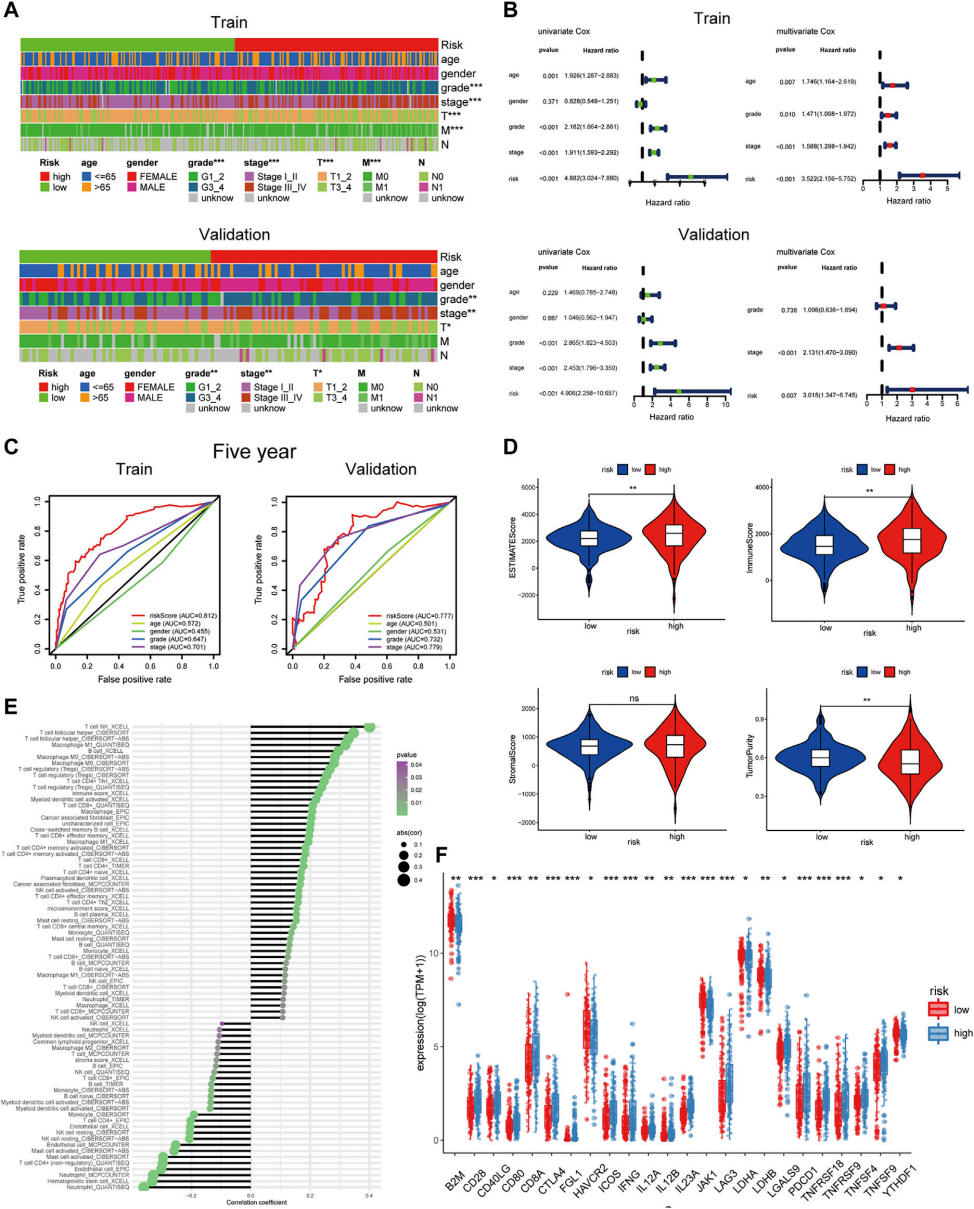
Clinical characteristics and immune infiltration of risk groups based on riskScore. **(A)** The heatmap reveals the distribution of clinical characteristics in patients in the high- and low-risk groups. **(B)** Univariate and multivariate Cox regression analyses showed that risk was an independent prognostic factor. **(C)** The receiver operating characteristic curve of riskScore and clinical features. **(D)** Different levels of estimate score, immune score, stromal score, and tumor purity in different risk groups. **(E)** Lollipop chart of tumor-related infiltrating immune cells on the basis of TIMER, CIBERSORT, CIBERSORT-ABS, QUANTISEQ, MCPCOUNTER, XCELL, and EPIC algorithms in different risk groups. **(F)** The expression levels of common immune checkpoints in different risk groups (***p < 0.001, **p < 0.01, *p < 0.05).

**FIGURE 8:**
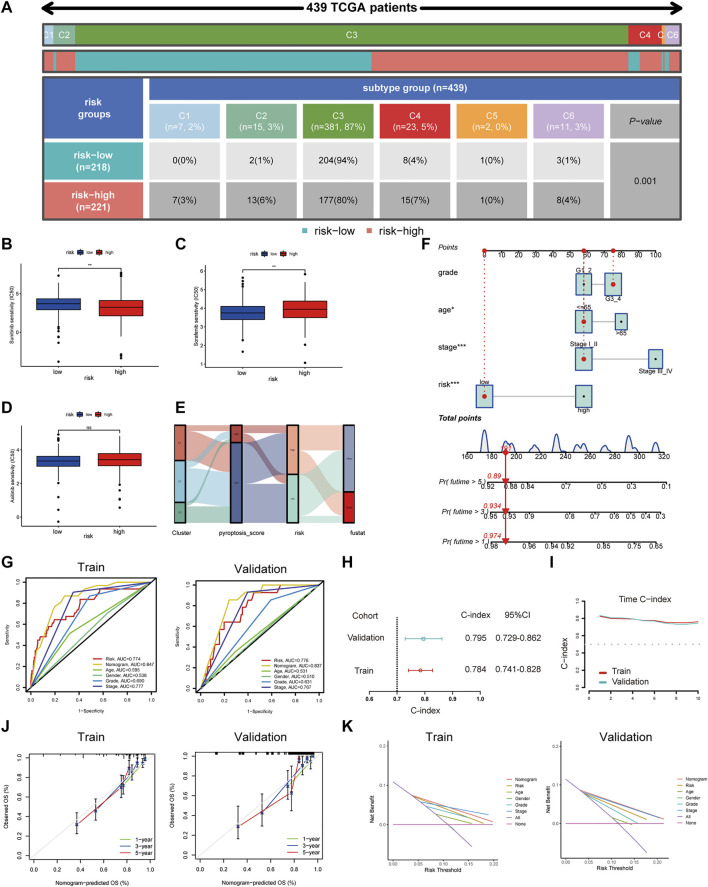
Drug treatment response and nomogram construction and validation. **(A)** The distribution of six immune subtypes of patients in high- and low-risk groups. **(B–D)** Different IC50 of targeted drugs between the patients in high- and low-risk groups. **(E)** The alluvial diagram demonstrated the relationship between risk groups based on riskScore and molecular subtypes. **(F)** A nomogram containing clinical features and the risk groups based on riskScore to predict survival time. **(G)** The receiver operating characteristic curve of nomogram and clinical features. **(H)** The C-index for exhibiting the ratio of the predicted result to the actual result. **(I)** The C-index trend over time. **(J)** The calibration curve was used to predict the OS of ccRCC patients in the training and validation cohorts. **(K)** The NB assessing the outcome was illustrated by DCA.

To explore the clinical value of the risk groups, we plotted a survival curve to evaluate the survival of patients in the low- or high-risk groups. The outcomes showed that, relative to the patients in the low-risk groups, the prognosis of patients in the high-risk groups was worse in both the training and validation sets (*p* < 0.001, Figure 6F). According to the riskScore and survival status of patients, we noted an increase in the mortality rate of patients with high riskScore (Figure 6G). To explore whether the risk model was suitable for different clinical groups, we merged the riskScore and clinical information from the training and validation sets and sketched the survival curves of grades and stages at different stages. The outcomes showed that, compared to the patients in the low-risk groups, the prognosis of patients in the high-risk group was worse regardless of the grade and stage (*p* < 0.001, Figure 6H). Subsequently, we explored the relationship between riskScore and clinical characteristics. The heatmap displayed the distribution of age, gender, stage, and grade in the training and validation sets. It could be seen that riskScore was significantly correlated with the grade, stage, T, and M in the training set (*p* < 0.05) and grade, stage, and T in the validation set (*p* < 0.05, Figure 7A). The boxplots presented the relationship between the clinical characteristics and riskScore (Supplementary Figure S5A,B). External validation on the GEO dataset also confirmed that patients with stage III_IV had a higher riskScore (Supplementary Figure S5C). The univariate Cox regression analysis showed that risk groups based on riskScore were essential risk factors for ccRCC (HR > 1, *p* < 0.001). Furthermore, multivariate Cox regression analysis showed that it was also an independent prognostic factor of ccRCC (Figure 7B). The ROC curves indicated that, compared with other factors, riskScore exhibited better prediction ability in 5 years (Figure 7C and Supplementary Figure S6A). It meant that riskScore performed better in predicting long-term survival of patients compared with various clinical indicators. These outcomes indicated that the risk model could be applied as a vital indicator for evaluating the prognosis of ccRCC, which was confirmed in the validation set. Then we further investigated the correlation with the immune microenvironment of the signature which was based on PRlncRNAPs. Patients in the high-risk group had lower tumor purity and higher immune score than the low-risk group, but no difference in stromal score (Figure 7D). The lollipop chart presented that riskScore was positively correlated with Tregs, cancer-associated fibroblasts, T follicular helper cell, memory-activated CD4^+^ T cell, and macrophage M0 and negatively correlated with NK cell resting, mast cell activated, etc. (Figure 7E). Differential analysis of immune checkpoints suggested that the expressions of CTLA4, PDCD1, LAG3, and TNFRSF4 were upregulated in high-risk patients, but JAK1 and HAVCR2 were downregulated (Figure 7F). Vesteinn Thomson’s study (Thorsson et al., 2018) indicated that tumors were divided into six immune subtypes, including wound healing (C1), IFN-γ dominant (C2), inflammatory (C3), lymphocyte depleted (C4), immunologically quiet (C5), and TGF-β dominant (C6). Among the six immune subtypes, C3 has the best prognosis. Although there were many immune components, the prognosis of C1 and C2 was still poor. C4 and C6 encompassed mixed immune characteristics and underwent the worst prognosis. C3 accounted for the highest proportion of ccRCC patients, accounting for 87%. Relative to the patients in the low-risk group, the proportion of C3 subtypes with better prognosis was notably decreased in the high-risk group (high-risk group 80%, low-risk group 94%, *p* < 0.05), and C1, C2, C4, and C6 subtypes with poor prognosis were increased (Figure 8A). Concerning drug treatment, the IC50 of sunitinib in the high-risk group was lower (Figure 8B), while the IC50 of sorafenib was higher (Figure 8C), but no difference in axitinib (Figure 8D). Finally, the alluvial diagram presented the distribution of pyroptosis subtypes, pyroptosis-score, and risk groups based on riskScore (Figure 8E).

### Construction of Nomogram to Predict Survival

Considering the clinical practicability, we established a nomogram combining risk groups based on riskScore and clinicopathological parameters to predict 1-, 3-, and 5-year survival (Figure 8F). The predictive parameters were the results of our previous independent prognostic analysis, containing risk, stage, grade, and age. The AUCs of the nomogram were 0.847 and 0.837 (Figure 8G) and the C-indexes were 0.784 and 0.795, respectively, in the training and validation sets (Figure 8H). Time C-index showed that C-index changed over time and was maintained at about 0.8 (Figure 8I). Therefore, our nomogram was dependable in forecasting the prognosis of ccRCC (Figure 8J). Next, an ROC curve was plotted to observe the predicted value of the nomogram with or without the risk. The AUC displayed corresponding improvements in the training and validation sets after adding the risk to the predictive model (Supplementary Figure S6B). DCA results illustrated the net benefit (NB) assessing the ccRCC patients’ outcomes by employing the risk groups based on riskScore, tumor stage, age, gender, grade, or a combination of some features (clinicopathological parameters in nomogram). The results showed that combining the risk groups based on riskScore with tumor stage, grade, and age significantly increased the NB (Figure 8K). Finally, based on the model built on the training set, we integrated the data of the TCGA training set and the validation set and compared it with the previous model. The results showed that our model had higher AUC value (Supplementary Figure S7A) and C-index (Supplementary Figure S7B).

### Validation of the Pyroptosis-Related lncRNA

In our study, we established a prognostic model of eight PRlncRNAPs for predicting the prognosis of ccRCC. To further screen PRlncRNA targets, we intersected eight pairs of lncRNAs (13 lncRNAs) with differential genes in ccRCC and obtained five lncRNAs (Supplementary Figure S8A). The heatmap showed the expression of five lncRNAs in ccRCC and noncancerous tissues (Supplementary Figure S8B). Subsequently, we performed co-expression analysis of the above five lncRNAs with 19 DEPRGs, and got an lncRNA with the highest correlation, AC002331.1, also called LINC02195 (|correlation coefficient| > 0.6, *p* < 0.001, Supplementary Table S6). Then, we analyzed the expression of LINC02195 in different grades and stages of ccRCC by UALCAN database. The results suggested that compared to normal condition, all four stages and grades highly expressed LINC02195, showing a statistical significance except stage1 (*p* < 0.05, Supplementary Figure S8C,D). In the subtype analysis of ccRCC, we found that the expression level of LINC02195 was higher in ccB-type patients than ccA (Supplementary Figure S8E). The survival curves indicated that high expression of LINC02195 meant worse survival (Supplementary Figure S8F). Pan-cancer analysis displayed that LINC02195 was significantly upregulated in multiple tumors (Supplementary Figure S9A). Then, we detected the expression of LINC02195 in human renal cortical proximal tubule epithelial cell line HK2 and human renal clear cell carcinoma cell lines (786-O, 769-P, CAKI-2, OS-RC-2), and we found that LINC02195 in renal clear cell carcinoma cell line was significantly higher than that in HK2 (Supplementary Figure S9B). Finally, LINC02195 was highly expressed in 32 renal clear cell carcinoma tissues than matched adjacent tissues (Supplementary Figure S9C).

## Discussion

Pyroptosis is a kind of programmed cell death. Unlike apoptosis, pyroptosis is an inflammatory death of cells. When pyroptosis occurs, cells will release inflammatory mediators, which trigger the body’s inflammatory response (Liu X. et al., 2021b). More and more studies have proved that pyroptosis may perform a dual function of stimulating or preventing cell growth or death in different tumor cells (Xia et al., 2019). Pyroptosis can promote tumor death to restrain the development of cancer. On the other hand, cells will activate numerous signaling pathways and release a large number of inflammatory mediators when pyroptosis occurs, which are associated with the occurrence and drug resistance of tumors (Zhou and Fang, 2019).

Previous studies have usually focused on a single pyroptosis molecule or a single type of tumor microenvironmental cell. Recently, attention has been paid to the effects of multiple genes on multiple tumor phenotypes. For example, Ye’s study investigated the roles of numerous PRGs in colorectal cancer, and such analysis models allow to explore pyroptosis’ function in cancer from a whole point of view (Song W. et al., 2021). There have also been studies discussing the role of pyroptosis in ccRCC, like Zhang’s research (Zhang X. et al., 2021). They classified ccRCC into four types based on the expression of PRGs. Both subtypes B and C with high PRG expression were enriched in immune cells, but showed two different outcomes of good prognosis and poor prognosis. Zhang speculated that intricate cytokines secreted by cancer cells were the reason for this difference. Different from Zhang’s study, we divided ccRCC into three subtypes based on the PRG expression after a comprehensive and systematic analysis. Among them, immune cells were significantly enriched and many immunosuppressive cells were activated in C2 and C3 with high PRG expression, both of which showed poor prognosis. The results of C1 with low PRG expression are opposite to C2 and C3. The results of our analysis were more uniform, which better explained the role of pyroptosis in mediating immune escape.

Among the three subtypes of pyroptosis we identified, it was found that activated dendritic cell, MDSC, macrophage, activated CD4 T cell, activated CD8 T cell, T follicular helper cell, and natural killer T cell were more infiltrated in C2 and C3 subtypes with poor prognosis, and there were more Tregs in C3 subtype. It has been reported that CD8^+^ T cells infiltrated in RCC are in a state of disability and promote the formation of immune escape (Dai et al., 2021). Treg is a type of T cell with a significant immunosuppressive effect, which can inhibit the immune response of other cells (Hatzioannou et al., 2021). Therefore, it implied that pyroptosis may recruited immune cell infiltration and established an interference and inhibition state in the TME according to previous study and our results.

Immune checkpoints are a class of immunosuppressive molecules that express in immune cells and regulate the degree of immune activation, which prevents autoimmunity, but at the same time may also be the cause of tumor immune escape (Giraldo et al., 2015). Our results revealed that the expression of CTLA4, PDCD1, and PDCD1LG2 in patients with C2 and C3 subtypes was higher than that in C1. Cytotoxic T lymphocyte-associated antigen 4 (CTLA4 or CD152) is an immune checkpoint that negatively regulates T-cell-mediated immune responses by intrinsic and extrinsic mechanisms. CTLA4 delivers a negative signal to effector T cells directly and was mainly associated with functions of Tregs (Lisi et al., 2021). Both PDCD1 (also known as human PD-L1) and PDCD1LG2 (also known as human PD-L2) are ligands of PD-1 (also known as CD274), which inhibit the function of T lymphocytes by binding with PD-1, thereby inhibiting the autoimmune response (Nunes-Xavier et al., 2019). The results of the above analysis showed that the pyroptosis-activated state could regulate the expression of various immune checkpoints, inhibit the functions of various immune cells, and interfere with the clearance of tumors by immune cells, which further indicated that pyroptosis can mediate immune escape of tumors. In order to predict the effect of drug treatment in patients with different subtypes, we constructed the immunophenogram for forecasting anti- PD-1 therapy sensitivity of ccRCC, which presented that C3 patients were in an activated state of pyroptosis and were sensitive to anti-PD-1 therapy and a combination of anti-CTLA4 and anti-PD-1 therapy. However, the outcomes need further verification in the future. The drug treatment response showed that patients in the activated state of pyroptosis were more sensitive to sorafenib and sunitinib. GSVA enrichment analysis, GO, and KEGG analysis of common differential genes between pyroptosis subtypes illustrated that the enrichments of immune-related biological processes in the pyroptosis-activated state were more significant. In a word, targeted pyroptosis may reverse the immune interference and suppression state, thereby enhancing the effect of immunotherapy.

In order to further analyze the regulation of pyroptosis on immunity, we constructed a pyroptosis-score by performing PCA on common differential genes associated with prognosis in the three pyroptosis subtypes. We found that memory-activated CD4 T cell, T follicular helper cell, Tregs, and macrophage M0 cells were more infiltrated in patients with high pyroptosis-score, and pyroptosis-score was positively correlated with immune checkpoints CTLA4, PDCD1, and PDCD1LG2. The above results further illustrated that pyroptosis played an indispensable role in regulating immune activity and mediating tumor immune escape. Patients with high pyroptosis-score were more responsive to sorafenib and sunitinib, which indicated that patients with pyroptosis activation may benefit more from these two targeted therapy agents.

Nowadays, more and more functions of lncRNAs have been discovered (Statello et al., 2021), such as regulating gene expression, posttranscriptional modification, and splicing. It was reported that lncRNAs could regulate the pyroptosis in various tumors, and the researchers also tried to explore the regulatory role of lncRNAs on pyroptosis in ccRCC. For example, one study explored and established a prognosis model of PRlncRNAs (Tang et al., 2021). However, owing to the differences in data processing among different data sets, such a model cannot compare the absolute expression levels of lncRNA well. Therefore, we established a PRlncRNAPs prognostic model which can eliminate differences in data processing and explore its ability to forecast the survival of ccRCC patients. We developed a riskScore based on PRlncRNAPs and divided the patients into high- and low-riskScore groups. Survival analysis indicated that patients with higher riskScore have a worse prognosis. Clinical correlation analysis showed that riskScore was correlated with stage, grade, T, and M, which indicated that the riskScore has perfect prognostic value and clinical value. There were more immune cells infiltrated in patients with high riskScore, such as Tregs, cancer-associated fibroblasts (CAF), T follicular helper cell, memory-activated CD4^+^ T cell, and macrophage M0 cells, which was consistent with pyroptosis subtypes and pyroptosis-score. Concerning the three targeted drug treatments, patients with high riskScore were more sensitive to sunitinib but less sensitive to sorafenib than patients with low riskScore. However, there was no difference with axitinib. Finally, we integrated various prognosis-related indicators to establish a nomogram. The ROC curve, calibration curve, DCA, and C-index were applied to confirm that the prognostic model can faultlessly forecast OS.

To further screen PRlncRNA targets, we found an lncRNA, LINC02195, which was intensely associated with 19 DEPRGs from eight pairs of lncRNAs related to prognosis by co-expression analysis. Pan-cancer analysis and survival analysis indicated that it was upregulated in various tumors and resulted in poor prognosis. qRT-PCR confirmed that it was highly expressed in ccRCC tissues and cells, providing a theoretical basis for mechanism exploration.

In conclusion, we performed comprehensive and systematic bioinformatics analysis to deeply analyze the function of pyroptosis in ccRCC, distinguished three pyroptosis subtypes, and constructed a PRlncRNAPs model. Pyroptosis activation meant worse prognoses and infiltration of more immunosuppressive cells that was conducive to tumor immune escape and tumor progression. We also predicted its response to current first-line treatment drugs, providing new ideas for clinically guiding ccRCC patients’ personalized immune and targeted therapy strategies. In addition, we developed a constructive and feasible prognostic model based on eight PRlncRNAPs, which performed well in forecasting the prognoses of ccRCC patients and assessing immune cell infiltration in ccRCC. However, several unidentified mechanisms remain to be explored for the interaction between pyroptosis and lncRNAs. Finally, we hope that the analysis of pyroptosis and lncRNAs in this study can provide new strategies for individualized therapy and immunotherapy of ccRCC.

## Abbreviations

AUC, area under the curve; DEGs, differentially expressed genes; ccRCC, clear cell renal cell carcinoma; DEPRGs, differentially expressed pyroptosis-related genes; TME, tumor microenvironment; GSVA, gene set variation analysis; GO, Gene Ontology; HR, hazard ratio; lncRNA, long noncoding RNA; KEGG, Kyoto Encyclopedia of Genes and Genomes; LASSO, least absolute shrinkage and selection operator; OS, overall survival; PCA, principal component analysis; ssGSEA, single-sample gene set enrichment analysis; PRlncRNAPs, pyroptosis-related long noncoding RNA pairs; ROC, receiver operating characteristic.

## Data Availability

The data sets presented in this study can be found in online repositories. The names of the repository/repositories and accession number(s) can be found in the article/Supplementary Material.
