# Supplementary material for: Analysis of Pyroptosis-Related Immune Signatures and Identification of Pyroptosis-Related LncRNA Prognostic Signature in Clear Cell Renal Cell Carcinoma
Source: Front Genet. 2022 Jun 29;13:905051. doi: 10.3389/fgene.2022.905051 (PMC9277062; doi:10.3389/fgene.2022.905051)
Supplement: Supplementary file 1 [file DataSheet1.docx]

**Supplementary Materials**

**Supplementary Figures**

**Supplementary Figure 1.**

**
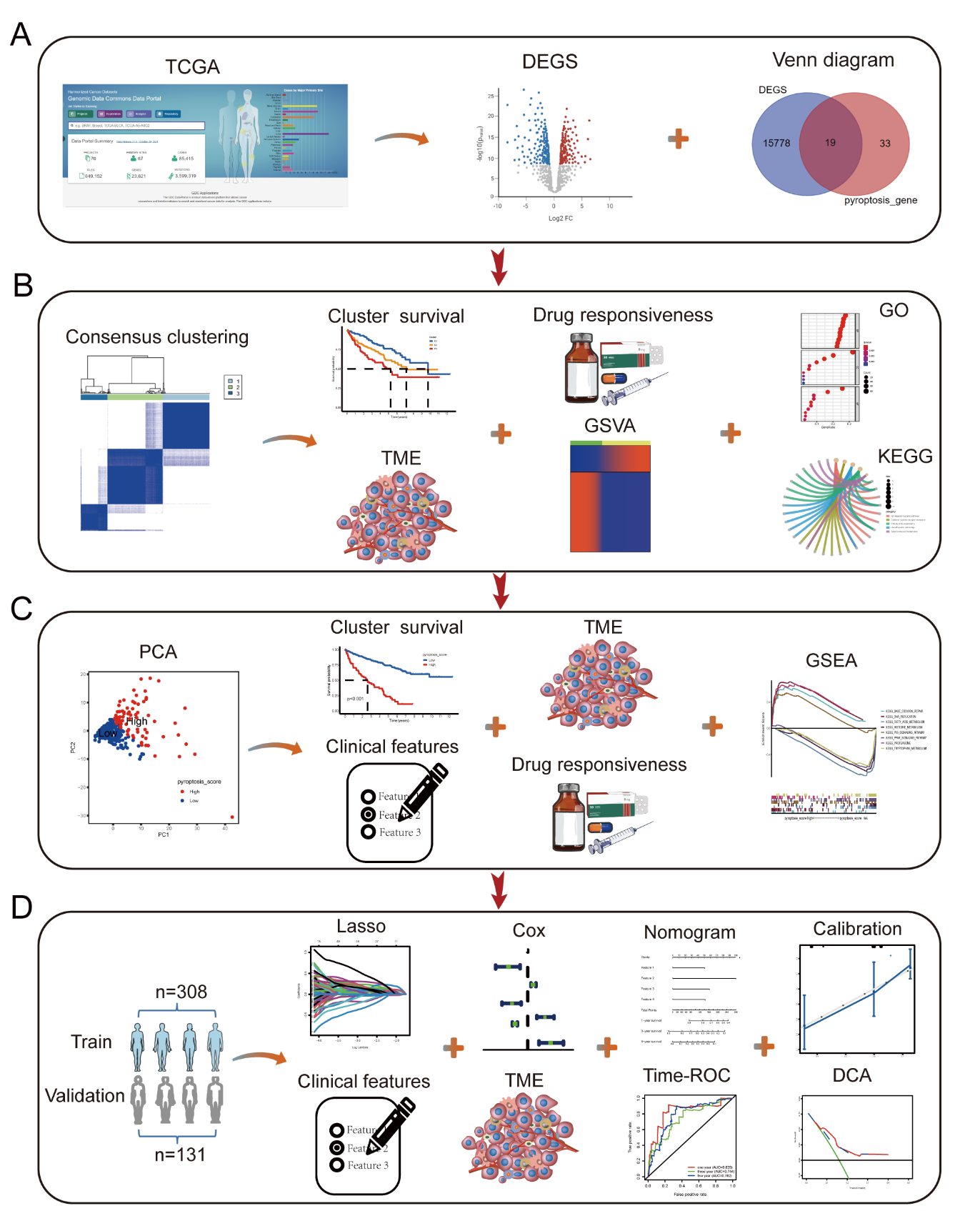
**

**Supplementary Figure 1.** Flow chart depicting the main process of analysis. (A) Identification of 19 DEPRGs by differential analysis. (B) Consensus clustering analysis of DEPRGs and the differences of the tumor microenvironment, immune infiltration, and drug therapy among pyroptosis subtypes. (C) Construction of pyroptosis-score and the relationship with TME and drug response. (D) Construction of OS-Related PRlncRNAPs Model and the clinical value of risk groups.

**Supplementary Figure 2.**

| **** | **** | | **** | | **** | | **** |
| --- | --- | --- | --- | --- | --- | --- | --- |
| **** | **** | | **** | | **** | | **** |
| **** | | **** | | **** | | **** | |

**Supplementary Figure 2.** Survival analysis curves of 14 DEPRGs related to prognosis.

**Supplementary Figure 3.**

| **** | **** | | **** | | **** |
| --- | --- | --- | --- | --- | --- |
| **** | | **** | | **** | |

**Supplementary Figure 3.** Survival analysis curves of the immune checkpoint, p<0.05.

**Supplementary Figure 4.**

**
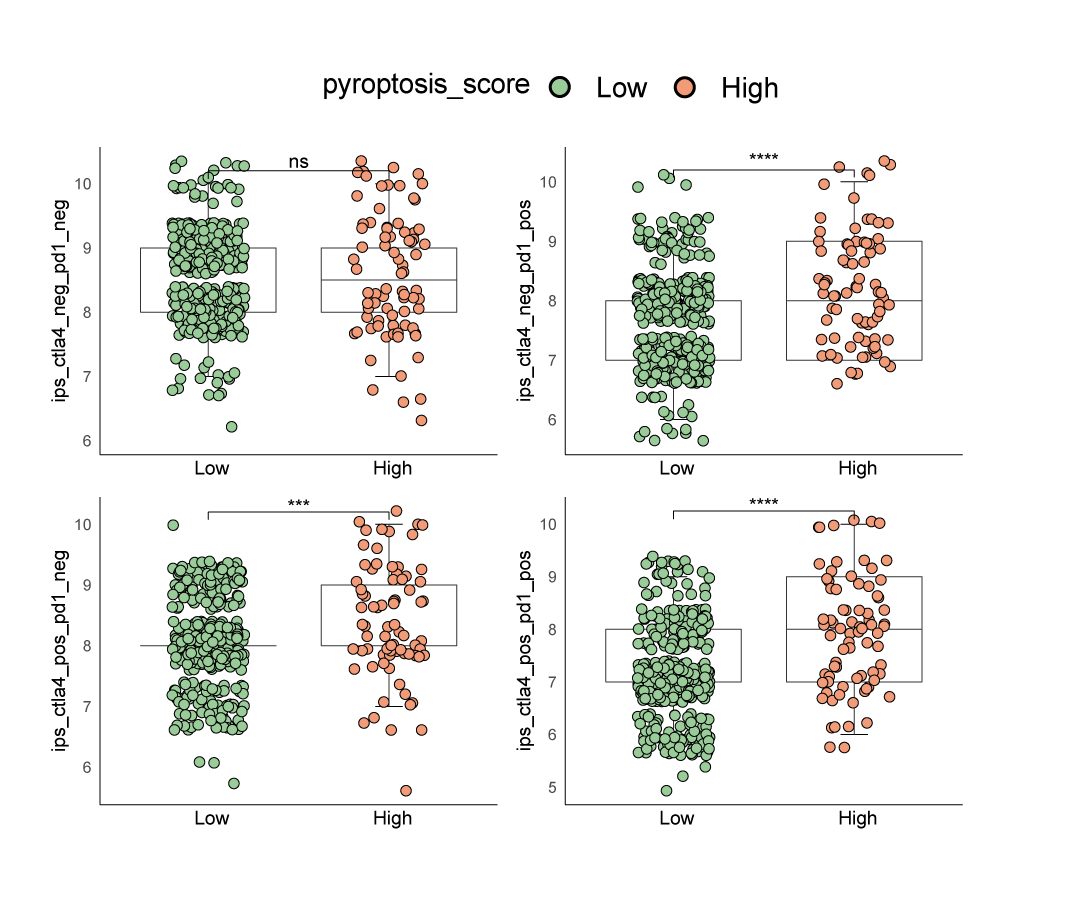
**

**Supplementary Figure 4.** IPS comparison among high and low pyroptosis-score of the ccRCC patients in the CTLA4 negative/positive or PD-1 negative/positive groups. CTLA4_positive or PD-1_positive respectively stood for anti-CTLA4 or anti-PD-1 therapy (***P < 0.001, **P < 0.01, *P < 0.05).

**Supplementary Figure 5.**

**
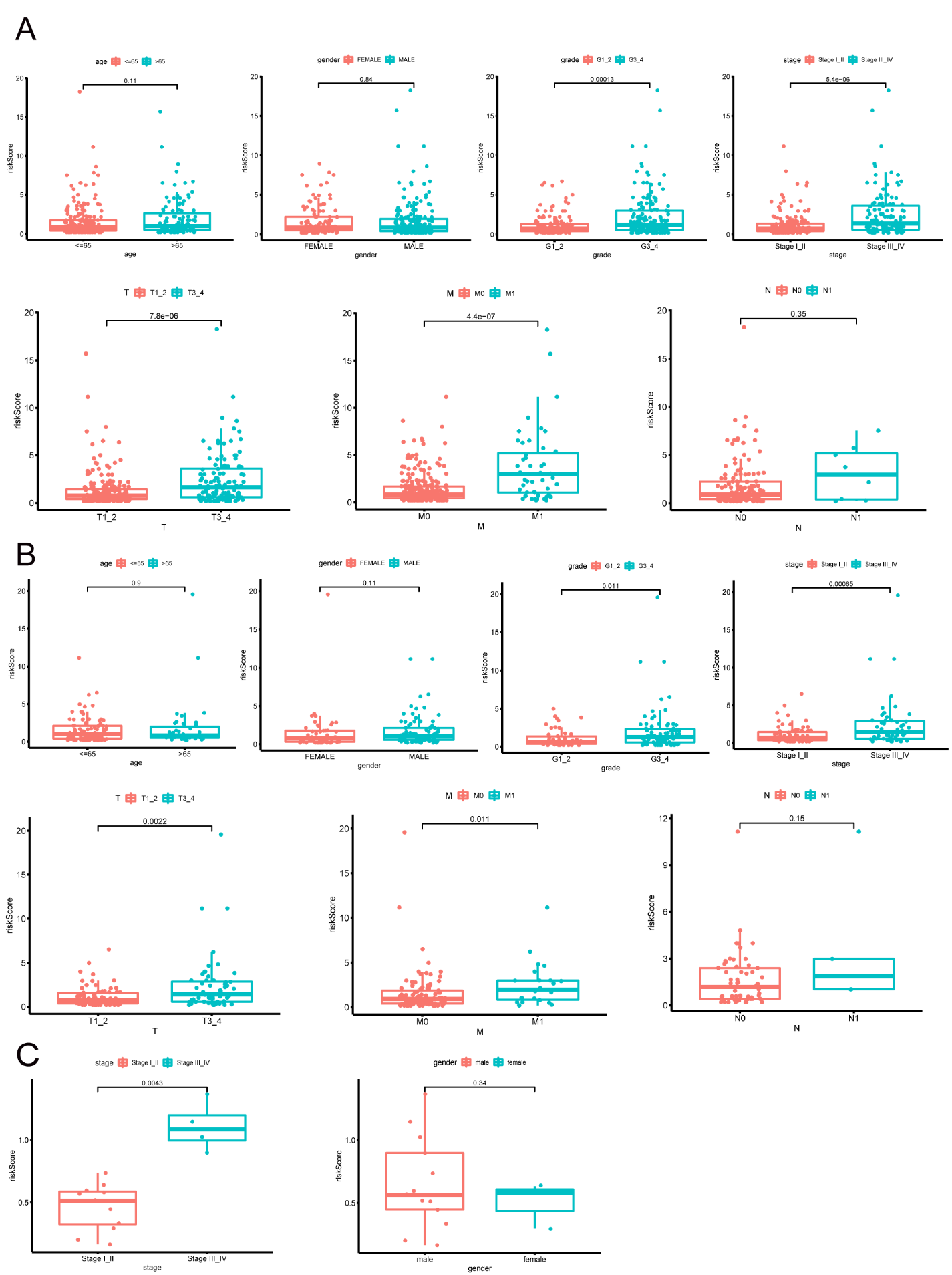
**

**Supplementary Figure 5.** The clinical characteristics associated with riskScore. Clinical subgroup analysis of riskScore in TCGA training set (A), validation set (B) and GSE76207 (C).

**Supplementary Figure 6.**

**
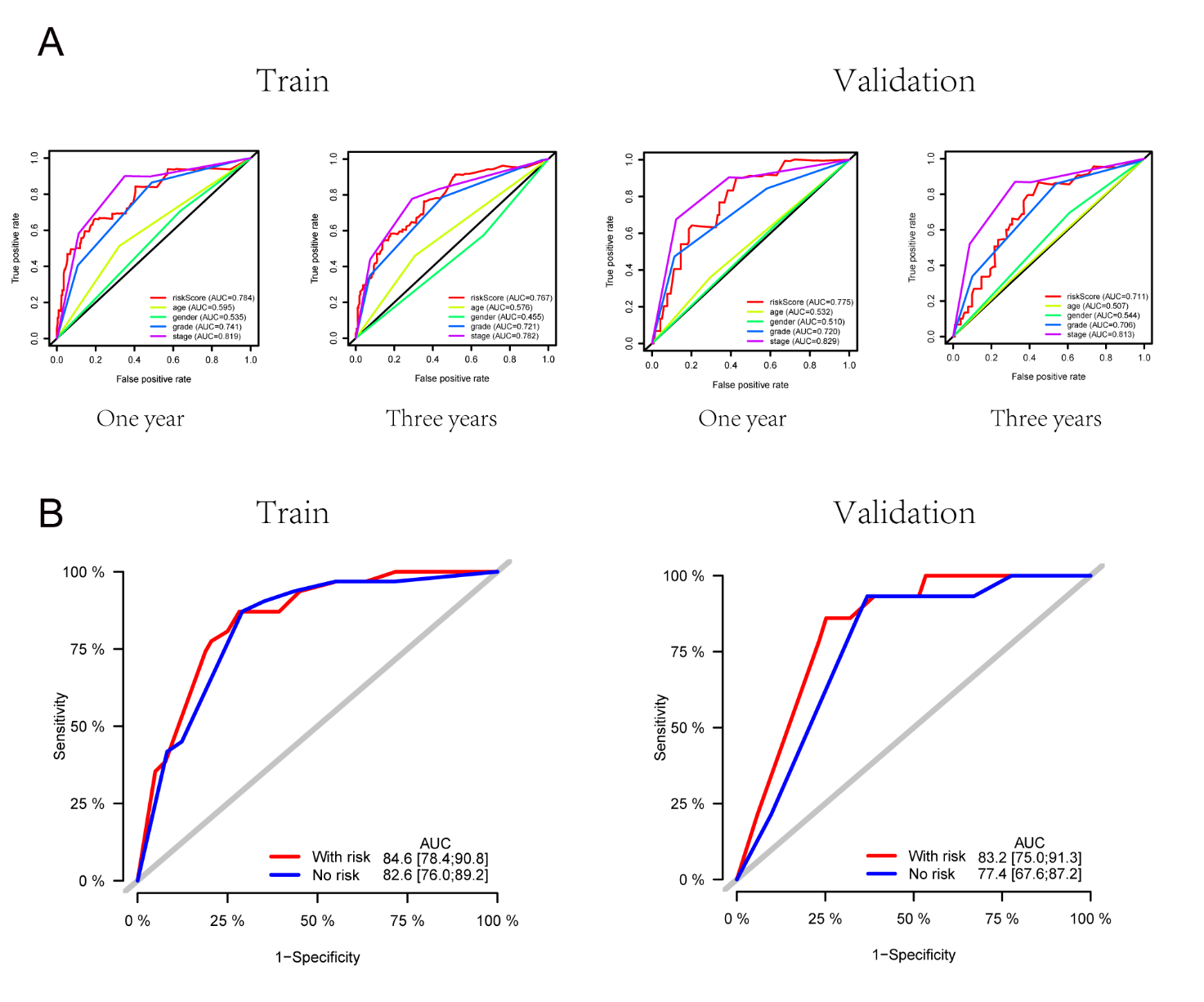
**

**Supplementary Figure 6.** The ROC curve evaluated the model's ability to predict prognosis. (A) The ROC curves of riskScore and clinical characteristics. (B) ROC curve analysis of OS predictions by the nomogram with or without risk.

**Supplementary Figure 7.**

**
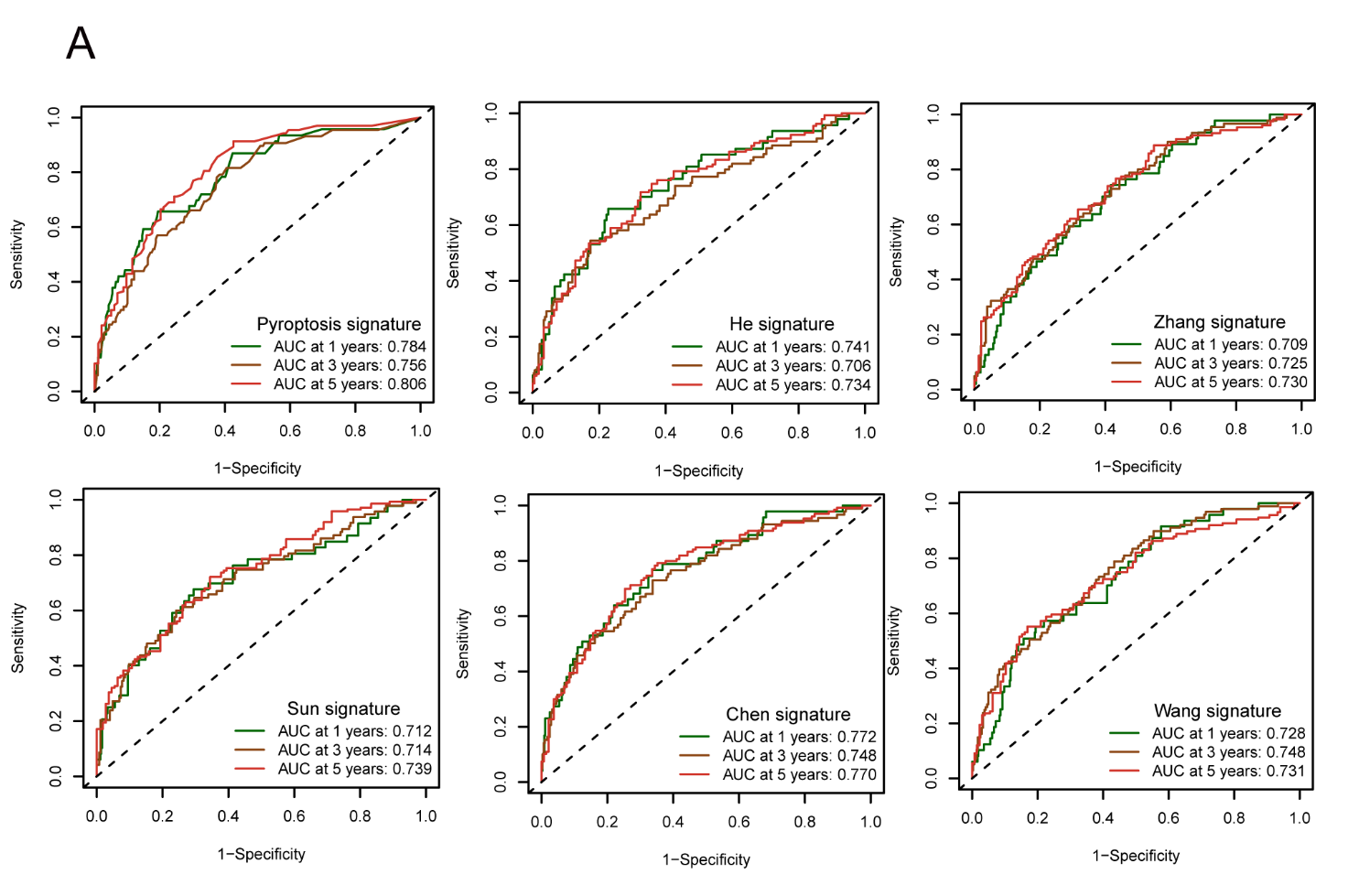
**


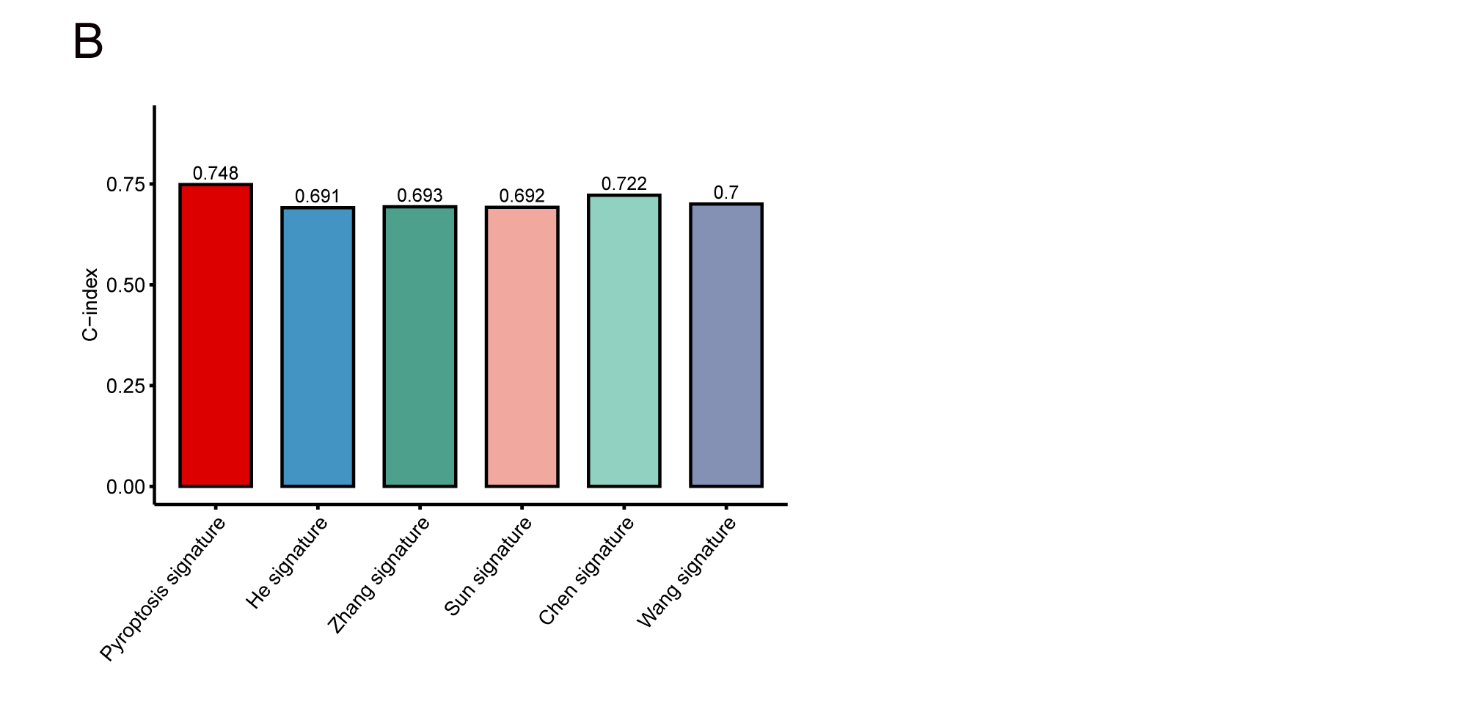


**Supplementary Figure 7.** Comparison of our prognostic model with other investigators' models. (A) ROC curves comparing the ability of different models to OS predictions. (B) Comparison of C-index of different models.

**Supplementary Figure 8.**

**
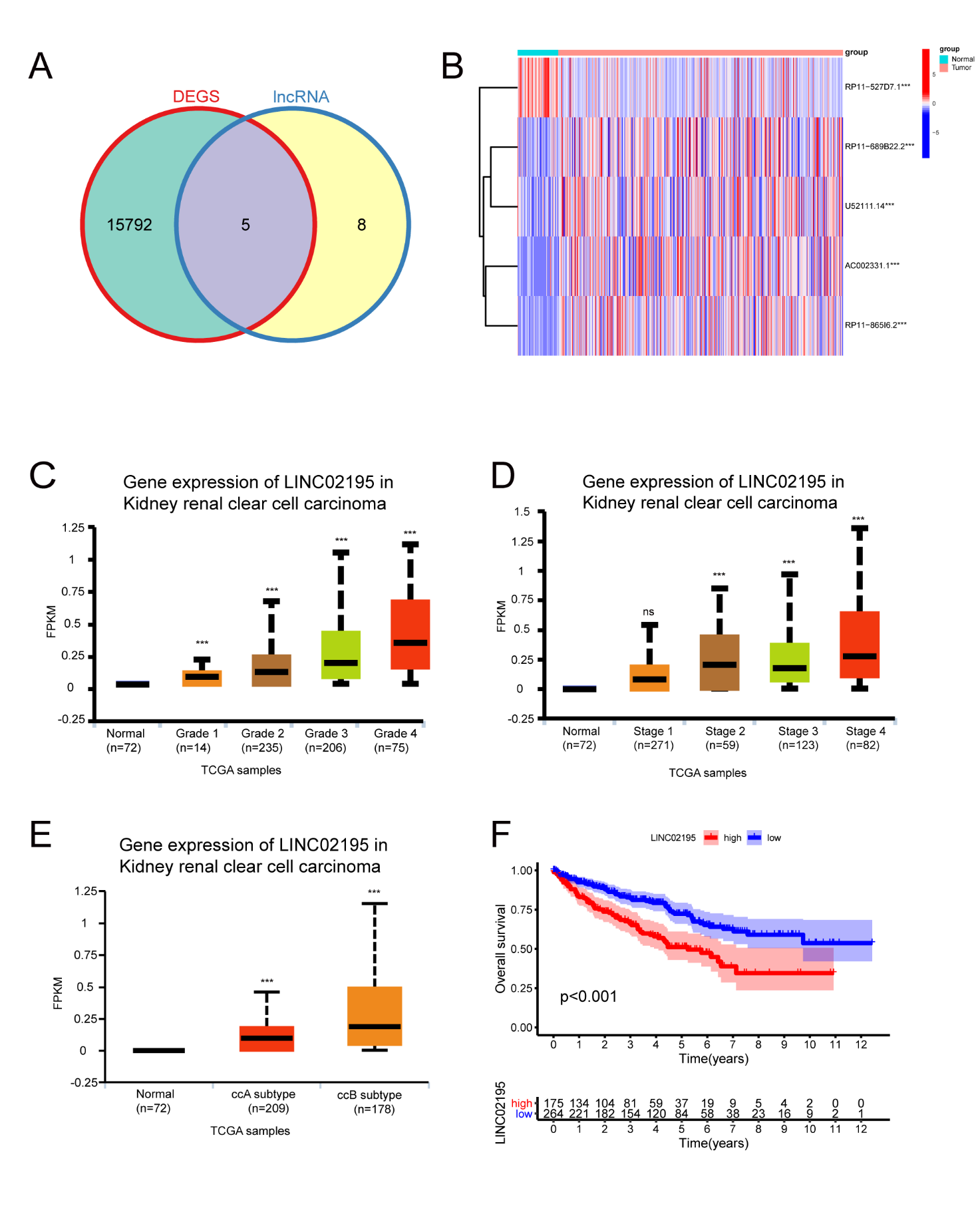
**

**Supplementary Figure 8.** Identifying the key gene of pyroptosis-related lncRNA. (A) The intersection of differential genes in ccRCC and 8 PRlncRNAPs. (B) A heatmap showed the expressions of 5 differentially expressed LncRNAs in ccRCC and non-cancerous tissues. (C, D) The expression of LINC02195 in different grades(C) and stages(D) of ccRCC. (F) The expression of LINC02195 in different subtypes of ccRCC. (F) Survival analysis of LINC02195 (***P < 0.001, **P < 0.01, *P < 0.05).

**Supplementary Figure 9.**

**
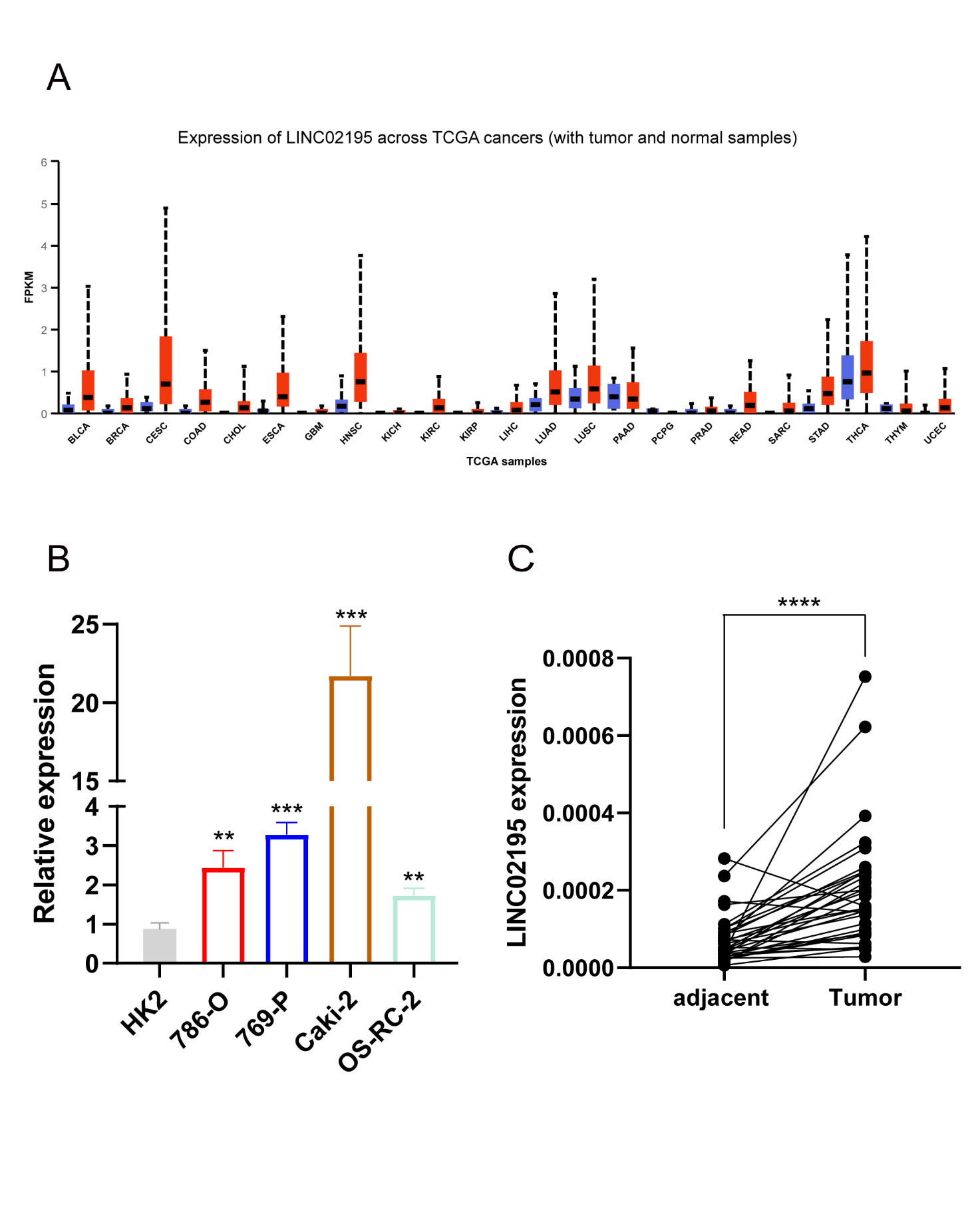
**

**Supplementary Figure 9.** The expression of LINC02195 in tissues and cells. (A) UALCAN analysis of the expression level of LINC02195 across TCGA cancers (with tumor and normal samples). (B) Expression levels of LINC02195 in HK2 and human renal clear cell carcinoma cell lines (786-O, 769-P, Caki-2, OS-RC-2). (C) Expression levels of LINC02195 in 32 renal clear cell carcinoma tissues and matched adjacent tissues (****P < 0.0001, ***P < 0.001, **P < 0.01, *P < 0.05).
